# Improving Immunotherapy Efficacy in Soft-Tissue Sarcomas: A Biomarker Driven and Histotype Tailored Review

**DOI:** 10.3389/fimmu.2021.775761

**Published:** 2021-12-03

**Authors:** Matthieu Roulleaux Dugage, Elise F. Nassif, Antoine Italiano, Rastislav Bahleda

**Affiliations:** ^1^ Département d’Innovation Thérapeutique et des Essais Précoces (DITEP), Gustave Roussy, Université Paris Saclay, Villejuif, France; ^2^ Département d’Oncologie Médicale, Institut Bergonié, Bordeaux, France

**Keywords:** immunotherapy, combination (combined) therapy, tertiary lymphoid structure (TLS), soft tissue sarcoma (STS), PD1 and PDL1

## Abstract

Anti-PD-(L)1 therapies yield a disappointing response rate of 15% across soft-tissue sarcomas, even if some subtypes benefit more than others. The proportions of TAMs and TILs in their tumor microenvironment are variable, and this heterogeneity correlates to histotype. Tumors with a richer CD8+ T cell, M1 macrophage, and CD20+ cells infiltrate have a better prognosis than those infiltrated by M0/M2 macrophages and a high immune checkpoint protein expression. PD-L1 and CD8+ infiltrate seem correlated to response to immune checkpoint inhibitors (ICI), but tertiary lymphoid structures have the best predictive value and have been validated prospectively. Trials for combination therapies are ongoing and focus on the association of ICI with chemotherapy, achieving encouraging results especially with pembrolizumab and doxorubicin at an early stage, or ICI with antiangiogenics. A synergy with oncolytic viruses is seen and intratumoral talimogene laherpavec yields an impressive 35% ORR when associated to pembrolizumab. Adoptive cellular therapies are also of great interest in tumors with a high expression of cancer-testis antigens (CTA), such as synovial sarcomas or myxoid round cell liposarcomas with an ORR ranging from 20 to 50%. It seems crucial to adapt the design of clinical trials to histology. Leiomyosarcomas are characterized by complex genomics but are poorly infiltrated by immune cells and do not benefit from ICI. They should be tested with PIK3CA/AKT inhibition, IDO blockade, or treatments aiming at increasing antigenicity (radiotherapy, PARP inhibitors). DDLPS are more infiltrated and have higher PD-L1 expression, but responses to ICI remain variable across clinical studies. Combinations with MDM2 antagonists or CDK4/6 inhibitors may improve responses for DDLPS. UPS harbor the highest copy number alterations (CNA) and mutation rates, with a rich immune infiltrate containing TLS. They have a promising 15-40% ORR to ICI. Trials for ICB should focus on immune-high UPS. Association of ICI with FGFR inhibitors warrants further exploration in the immune-low group of UPS. Finally translocation-related sarcomas are heterogeneous, and although synovial sarcomas a poorly infiltrated and have a poor response rate to ICI, ASPS largely benefit from ICB monotherapy or its association with antiangiogenics agents. Targeting specific neoantigens through vaccine or adoptive cellular therapies is probably the most promising approach in synovial sarcomas.

## Introduction

Immunotherapy of cancer has been the last major breakthrough in the fight against cancer (1, 2). New immune checkpoint inhibitors (ICI) target immune cells present in the tumor microenvironment (TME), namely T-lymphocytes with specific anti-tumor activity. These lymphocytes display an exhausted phenotype with inhibitory receptors (3). By antagonising these inhibitory signals, ICIs reactivate pre-existing anti-tumor immunity (4, 5) and effectively destroy tumor cells. This breakthrough is fairly recent, but the concept can be traced back to the late 19th century. William Coley was an orthopedic surgeon who efficiently treated limb sarcomas by injecting modified bacteria intratumorally (6). This treatment induced infections with inflammatory reactions followed by tumor regression. For decades, the immuno-oncology concept was thereafter investigated (7, 8) and ICIs have eventually proven only recently to prolong survival in patients with melanoma (9, 10), lung cancer (11, 12), urothelial carcinoma (13) and renal cancer (14), amongst others.

Sarcomas are rare tumors from mesenchymal origin, with an incidence of 5-6/100.000 habitants/year in western countries (15, 16). As precludes their origin, these tumors have very diverse and heterogeneous phenotypes, as mesenchymal cells can have osseous, cartilaginous, muscular or adipose differentiation for instance.

The latest World Health Organization classification reports over 120 sarcoma histotypes (17). These tumors are classically divided into bone tumors representing 15% of sarcomas and soft-tissue sarcomas (STS) representing 85% of cases (18). Gastrointestinal stromal tumor (GIST) is the most frequent sarcoma and the proto-typical example of efficient targeted therapy based on tumor molecular biology (19). Prognosis of STS, excluding the particular entity of GISTs, is dismal: 90% of patients will be diagnosed in the localized setting, 30-40% of STS will recur within five years after initial treatment, median overall survival after diagnosis of metastatic disease is roughly 20 months (20). However, prognosis is highly heterogeneous, depends on the histotype and other clinicobiological parameters such as age, sex, size of tumor and histopathological grade (21, 22).

As all rare and diverse diseases, these tumors benefit from centralized expert centers for treatment, as it prolongs survival (23, 24). Drug development in the field encounters one major obstacle: heterogeneity. For the last 40 years, no drug has been able to prove superior to anthracyclines in the first line setting (25). Phase 3 trials including all histotypes have consistently failed to prove their superiority in the general population of STS (26), although having promising phase 2 trials (27). Immunotherapy is no exception to this rule. Correct selection of patients is of paramount importance, through precise biomarkers and translational research (28–30).

A few methodological points need to be addressed in regard to clinical trials of immunotherapy in sarcomas. It is likely that the raw overall response rate (ORR) is not the optimal endpoint for such trials. On the one hand, the most effective drug used in STS is adriamycin. Although some histotypes yield higher response rate, this regimen has an ORR of roughly 10% as monotherapy in an unselected population (31) but allows stabilisation of disease and a median PFS of 6 months in advanced setting (25, 26, 31). In this light, it has been proposed that an active drug in STS yields a PFS rate of roughly 30-50% at six months in the first line and a PFS rate over 40% at three months in the second-line setting (32). On the other hand, response evaluation on purely volumetric evaluation such as RECIST does not efficiently represent biological efficacy of immune-based therapy. The best way to evaluate ICI efficacy and response is still a matter of debate (33–35). In fact, preliminary results of a neoadjuvant trial presented at ASCO 2020 in undifferentiated pleomorphic sarcomas (UPS) and dedifferentiated liposarcomas (DDLPS), has shown RECIST response was not correlated to pathologic response (36) and the discordance between pathologic response and radiographic response has also been noted in other tumor types with ICI (37).

Although the first cancer immunotherapy to report clinical benefit was in sarcomas, new ICIs have been somewhat disappointing in STS up to now. As displayed in **Table 1**, ICIs in the advanced setting in STS have consistently reported ORRs of roughly 15% with a median PFS around 3-4 months, when including all histotypes. Liposarcomas (LPS) and UPS are the most thoroughly studied histotypes, with slightly better response patterns than other subtypes. Leiomyosarcomas (LMS) and synovial sarcomas have consistently shown to be resistant to ICIs monotherapy. On the other hand, alveolar soft-part sarcomas (ASPS) seem to be particularly sensitive to these treatments.

**Table 1:** Trials of Immune Checkpoint inhibitors in advanced Soft-tissue Sarcomas.

Trial	Design	Molecule tested	Population/Histotypes	Overall Response Rate						Median progression free survival					
				Overall	LMS	LPS	UPS	Synovial sarcoma	ASPS	Overall	LMS	LPS	UPS	Synovial sarcoma	ASPS
**Trials for multiple histotypes**															
SARC028 (38)	Non-randomized, Phase 2 trial	Pembrolizumab 200mg flat dose q3w	Four cohorts of 10 patients in each histotype: LMS, DDLPS, UPS, synovial sarcoma	17.5%	0%	20%	40%	0%			15w	25w	30w	7w	
SARC028 expansion cohorts (39)	Non-randomized, Phase 2 trial	Pembrolizumab 200mg flat dose q3w	Two cohorts: 39 DDLPS and 40 UPS			10%	23%					2M	3M		
Alliance A091401 (40)	Multicentre, open-label, non comparative, Phase 2 trial	Nivolumab 3mg/kg q2w	42 STS	5%						1.7M					
		Nivolumab 3mg/kg + ipilimumab 1mg/kg q3w for 4 cycles followed by nivolumab 3mg/kg q2w	41 STS	16%						4.1M					
Alliance A091401 expansion cohorts (41)	Multicenter, open-label, non comparative, phase 2 trial	Nivolumab 3mg/kg q2w	15 DDLPS & 13 UPS			6.7%	7.7%					4.6M	1.5M		
		Nivolumab 3mg/kg + ipilimumab 1mg/kg q3w for 4 cycles followed by nivolumab 3mg/kg q2w	14 DDLPS & 14 UPS			14.3%	28.6%					5.5M	2.7M		
Somaiah et al. (36)	Non-randomized, Phase 2 trial	Durvalumab 1500mg + tremelimumab 75mg q4w for 4 cycles followed by durvalumab alone	57 soft-tissue sarcomas: 6 LPS, 5 UPS, 5 synovial sarcoma, 10 ASPS and others	14.3%						2.8M		2M	1.8M	7.46M	34.23M
**Trials for specific histotypes**															
Maki et al. (42)	Non-randomized, Two-stage, Phase 2 trial (terminated early due to lack of efficacy)	Ipilimumab 3mg/kg q3w	6 synovial sarcomas					0%						1.85 months	
Ben-Ami et al. (43)	Non-randomized, Two-stage, Phase 2 trial (terminated early due to lack of efficacy)	Nivolumab 3mg/kg q2w	12 uterine LMS		0%						1.8 months				
Blay et al. (44)	Non-randomized, Phase 2 trial	Pembrolizumab 200mg q3w	Rare sarcomas (incidence<0.2/100.000): 24 chordoma, 14 ASPS, 5 DSRCT, 6 SMARCA4-malignant rhabdoid tumors & 32 others	15%					35.7%	7.9 months					
OSCAR (45)	Single arm, Phase 2 trial	Nivolumab 240mg q2w	11 clear cell sarcomas and 14 ASPS	4%					7.1%	4.9 months					6 months
Gxplore-005 (46)	Single arm, Phase 2 trial	Geptanolimab 3mg/kg q2w	37 ASPS						37.8%						6.9 months

ASPS, Alveolar Soft Part Sarcoma; DDLPS, Dedifferentiated Liposarcoma; DSRCT, desmoplastic small round cell tumor; LMS, Leiomyosarcoma; LPS, Liposarcoma; UPS, Undifferentiated Pleomorphic Sarcoma.

A pooled analysis of nine anti-PD1/anti-PDL1 therapy trials in STS, found an ORR of 15.1% for STS as a whole (47). As a monotherapy, anti-PD1/anti-PDL1 ICIs were found to have an ORR of 18.7%. In combination therapies (including other immune-based therapies or anti-angiogenic therapies), anti-PD1/anti-PDL1 were found to have an ORR of 13.4%. However, these results are pooled results from distinct trials, with monotherapy and combination therapies. Specific histotypes might benefit from histotype-tailored combination therapies with antiangiogenics, chemotherapy or other ICIs.

In this review, we sought to define how to move immunotherapy forward in the sarcoma field. To do so, we first describe translational data regarding the immune tumor microenvironment (TME) of sarcomas, in order to define biomarkers of efficacy and resistance to ICI. Second, we address new potential alternative immune-based therapeutic options in order to increase immunotherapy efficacy. Finally, we describe translational data regarding specific major histotypes, in order to propose histotype-tailored approaches as next steps for clinical development.

For clarity reasons, we will focus only on STS. We did not include primitive neuro-ectodermic tumors (PNET), although 15% of them are STS in order to keep a more homogeneous line of conduct for this review. Immunotherapy and immunology of GISTs have been reviewed elsewhere (48). Kaposi sarcomas are a specific entity, notably regarding systemic immunity, and will not be included in the rest of this manuscript. Likewise, pediatric sarcomas have specific challenges, specifically regarding drug development and immunity.

## THE TUMOR MICROENVIRONMENT OF SOFT-TISSUE SARCOMAS

### Neoantigens and Mutational Load Across Soft-Tissue Sarcomas

Tumor mutational burden (TMB) is defined as the number of somatic mutations per coding area in a cancer genome. A higher TMB increases neoantigen expression, which allows the immune system to distinguish normal from cancer cells. This leads to an increased T cell reactivity (49), and eventually predicts response to ICIs (50).

In STS, a heterogeneous group of tumors, this question is all the most important. Providing the analysis of 100.000 tumor genomes, Chalmers et al. found a globally low tumor mutational burden across STS with a median of 2.5mut/Mb and only 5% of tumors harboring >20mut/Mb. This proportion depended once again on the histotype, and 13.4% of angiosarcomas had more than 20mut/Mb (51). In the TCGA cohort, the average mutational load was again low (1.06mut/Mb), whereas the genetic landscape of STS seemed characterized by a high number of copy-number alterations (CNA), often affecting MDM2-TP53 or p16-CDK4-RB1 pathways. Frequently mutated genes included TP53 (40 of 80 LMS), RB1 (LMS, UPS and MFS) as well as ATRX (52). The prognostic impact of tumor mutational burden remains unclear in STS, but based on the analysis of 68 localized STS, a middle mutational burden was associated with a poorer overall survival than a lower one (53). Notably, CNAs, which are predominant in STS, have been suggested to be less immunogenic than mutations in a pan-cancer study (54, 55). This particular immunogenicity difference regarding CNAs versus mutations merits more thorough evaluation specifically in STS.

Microsatellite instability is rare, representing around 2% of patients (56, 57), if not absent (58, 59) in STS.

The analysis of various histotypes allowed to discriminate simple genomics sarcomas, characterized by a clonal pathognomonic driver genomic alteration, from complex genomic sarcomas, which are usually characterized by a higher number of mutations and CNAs (60). For instance, synovial sarcoma is characterized by a t(X;18)(p11;q11) translocation and low mutational load, whereas UPS or LMS show no pathognomonic driver molecular alteration but a high number of CNAs and a higher TMB (see **Table 1**).

Interestingly, this model is correlated with TME: translocation-associated sarcomas are less infiltrated by tumor-associated macrophages than complex genomic STS and CD8+ lymphocytes are more abundant in the TME of CNA-driven sarcomas (61). Antigen presentation and PD-L1 expression are also associated with a more mutated profile, and translocation-associated sarcomas are rarely PD-L1 positive (62, 63). Furthermore, activated CD8+ T cells are particularly abundant in pleomorphic sarcomas, for which TMB is known to be higher (64).

Another approach could be to focus on special molecular alterations, responsible for an expression of tumor-associated antigens (TAAs) or neoantigens with a high affinity for MHC I complex. The question of the immunogenic impact of translocation-related and cancer-specific antigens is crucial. As an example, in synovial sarcomas, translocation-related peptides are known to show a high affinity toward HLA B7 and B27 (65). More recently, this approach has been facilitated by the creation of a tool to predict peptides affinity to MHC complexes (66).

Cancer-testis antigens (CTAs) are expressed in the testis, embryo and placenta, as well as in various malignancies, where they seem to trigger a specific T-cell response. Moreover, most of these CTAs are oncogenic, making them attractive targets (67). CTAs are expressed in STS (68), and three main groups are of interest: melanoma-associated antigen gene (MAGE), preferentially expressed antigen of melanoma (PRAME) and New York esophageal squamous cell carcinoma 1 (NY-ESO-1). Interestingly, their expression is not associated with complex genomics, but seems correlated to a hypomethylated genomic profile, thus underlining the rationale of associating epigenetic and immunotherapeutic approaches in STS (69). On the whole, around 20% and 12% of all STS respectively express NY-ESO-1 and MAGE-A4, possibly conferring them a better prognosis (70). Once again, this expression is histotype-dependent, and NY-ESO-1 is expressed in around 95% of MRLPS and 49-76% of SS (71–73), where they are promising therapeutic targets, versus respectively 0% and 9% of UPS and LMS (70). Interestingly, Iura et al. demonstrated in synovial sarcomas that CTAs were often coexpressed (51% of SS expressed NY-ESO-1 and MAGE-A4 and PRAME) and their expression was associated with a higher grade and a trend to poorer OS (74). Moreover, 41% of UPS show a high expression of MAGE-A3, which is significantly more important than in LMS, DDLPS and synovial sarcomas and seems to be associated with a richer lymphocyte infiltrate and higher HLA-A expression (75). To our knowledge, even if CTA expression seems associated with a richer immune infiltrate, their predictive value for response to ICIs has not been studied. However, they are well exploited therapeutic targets, as subsequently described.

### STS Are Variably Infiltrated With Immune Cells

A few studies focused on TME in STS, based on gene expression profiling. This TME is heterogeneous and correlates to histological subtype, even if TAMs and CD8+ T cells are generally the most represented cells (76–78).

Two main polarizations of TAMs are described in human cancers (79). M0 are non-activated macrophages. They differentiate to M1 when exposed to granulocyte-monocyte colony stimulating factor (GM-CSF), lipopolysaccharide (LPS) or interferon-gamma (IFN-γ) and they promote an inflammatory microenvironment through the expression of IL-1, IL-6, IL-12 or TNFα. On the other side, M2 differentiate from M0 macrophages in presence of M-CSF, IL-4 or IL-10 and they promote immune escape through a high expression of PD-L1, IL-10 or TGFβ. According to Dufresne et al., based on the RNA-sequencing of 253 tumor samples, monocyte/macrophage is the most important detected signature (76).

Hu et al. studied the expression of 364 genes related to the immune system in the TCGA (The Cancer Genome Atlas) cohort (80). Four clusters could be distinguished and if the expression of genes related to CD8+ T lymphocytes was variable and observed in only one group of tumors, the most expressed genes were consistently those related to macrophages, of predominantly M2 polarization, more immunosuppressive. This heterogeneity is corroborated by the study of Deng et al, based on transcriptomic analysis of 869 soft tissue sarcomas (81). Three immunological subtypes were characterized: group A was associated with an M2 macrophage-rich infiltrate and a lower density of memory CD4+ lymphocytes and mast cells. Group B had an infiltrate dominated by M0 (naive) macrophages and group C was associated with M1 polarization of the macrophages present, a richer infiltrate of CD8+ T cells and plasma cells. Gu et al. also analyzed the TCGA cohort and distinguished two prognostic groups, determined in particular by the density of the CD8+ T infiltrate and activated lymphocytes, and by the expression of pro-inflammatory cytokines (82).

NK cells are crucial in antitumor immunity, where they have an antigen and MHC I independent cytotoxic activity (83). They are present in sarcomas, independently of other tumor cells, and have a definite immunological impact: in the TCGA (The Cancer Genome Atlas) cohort, they are the only immune cell population correlated with survival based on RNA-sequencing signatures (52).

Petitprez et al. has recently developed a novel classification of STS, based on the composition of the TME in large cohorts of STS, using the MCP-counter method, which is an RNA-sequencing deconvolution method (84). Tumours were assigned to one of five Sarcoma Immune Classes (SICs), labelled A, B, C, D and E, with highly distinct profiles. Histological subtypes were distributed across all the SICs with leiomyosarcoma belonging mainly to SICs A and B. Three SICs showed homogeneous profiles. SIC A, “immune desert”, was characterised by the lowest expression of gene signatures related to immune cells, as well as low vasculature. SIC C, “vascularised”, was dominated by a high expression of endothelial cell-related genes. SIC E, “immune and TLS high”, was characterized by the highest expression of genes specific of immune populations such as T cells, CD8+ T cells, NK cells, cytotoxic lymphocytes. Strikingly, the strongest determinant of SIC E was the high expression of the B lineage signature (p=1.8e-29). SICs B and D were characterized by heterogeneous but generally “Immune low” and “Immune high” profiles, respectively. The B lineage signature, a hallmark of the immune-high subclass E, correlated with an improved survival of STS patients, in tumours with both high and low CD8+ T cells infiltration. The authors validated by immunohistochemistry the high density of B cells and presence of tertiary lymphoid structures (TLS) in the immune-high subclass of STS on an independent cohort. Tertiary lymphoid structures (TLS) are ectopic lymphoid structures that develop in tumors, and conduct the activation, proliferation and differentiation of effector and memory T and B lymphocytes.

Studies using a transcriptomic approach are corroborated by immunohistochemical staining in STS. Tumor-infiltrating lymphocytes (TILs) are present across all sarcoma subtypes. As described, their proportions are variable and depend on the histotype, and they are hampered by the lack of consensus to define positive cases in immunohistochemistry. TAMs are the most abundant cells in the microenvironment of STS, where they are more likely to be M2-polarized (61, 85). The highest M2 to total macrophages ratio was found in UPS (86). As previously mentioned, IL-10 secreted by M2-macrophages counterbalances the prognostic impact of B cell infiltrate. Furthermore, the population of CD8+ T cells in the TCGA cohort was negatively correlated to M0 and M2 macrophages, but positively associated with M1-macrophage infiltrate (87). Overall, 25-43% of STS are described as highly infiltrated in CD3+ T cells and a majority of them are CD8+ T cells (88, 89), outnumbering FoxP3+ regulatory T cells. In accordance to Petitprez et al, CD20+ cells are described in the TME of STS, where they are associated to T cell infiltration (85). Interestingly, their prognostic impact seemed counterbalanced by high IL-10 expression by CD163+ M2 macrophages, which underlines the crucial role played by tumor associated macrophages (TAMs) in the immunosuppressive environment of STS.

### Patterns of Immune Checkpoint Regulators in STS

As in many other tumor types, one of the main issues of PD-L1 expression assessing is the lack of reproducibility across tumor types: different clinical trials and anti-PD(L)1 drugs have led to different immunohistochemistry antibodies (22C3, SP142, 28-8…) and different thresholds for PD-L1+ tumors (>1%, 5%, 10%…) (90). Some other studies focus on RNA-sequencing, with a better reproducibility, but sometimes discordant results to immunohistochemistry.

PD-L1 is more expressed on TAMs than on tumor cells and seems associated with a PD-1+ and CD8+ T cell infiltrate (88). Pollack et al. provided a comprehensive analysis of PD-L1 expressing STS and their microenvironment, based on the study of the archival tumor samples of 81 patients (62). PD-L1 expression was correlated to CD8+ T cell infiltrate and antigen presentation gene expression (HLA-A and HLA-B). The infiltrating lymphocytes also showed a higher TCR Vβ clonality. Antigen presentation and PD-L1 expression were associated with a more mutated profile and translocation-associated sarcomas were rarely PD-L1 positive. Furthermore, it seems that PD-L1 driven immune escape occurs in tumors with an effective immune infiltrate characterized by the presence of Th1 CD4+ cells, B cells and dendritic cells, by a higher expression of type 1 and 2 MHC as well as by an activation of IFNα, IFNγ and tumor-necrosis factor alpha (TNFα) pathways (63). This immune escape is also associated with an increase in FoxP3+ regulatory T cells, which has a negative impact on prognosis (91). The underlying molecular mechanism of PD-L1 expression often depends on a copy-number gain (4 of 10 PD-L1+ cells) (92), which is associated with a higher mutational load. Previous treatments probably have an impact, and of 46 patients who received preoperative radiotherapy, 10.9% and 15.2% respectively showed an increase in PD-L1 expressing tumor cells and TAMs, thus underlining a potential synergy between anti-PD1/PD-L1 treatments and radiotherapy (93). No variation was observed in CD8+ and PD1+ T cells. PD-L1 expression is also limited by tumor heterogeneity, and there is a discordance in around 20% of cases between primary tumor and metastasis (94). In most cases (67%), PD-L1 was more expressed in metastasis than in the primary tumor, whereas CD8+ are to decrease in the relapse (95).

Besides transmembrane expression, an interesting study has assessed soluble PD-L1 (sPD-L1) levels in localized STS (96). sPD-L1 level was more associated with metastasis-free survival (MFS) and overall survival (OS) than PD-L1 tumor immunohistochemistry. No difference was found in sPD-L1 serum levels between tumors with PD-L1 positive and negative immunohistochemistry, which suggests that PD-L1 immune escape could also be driven by PD-L1 exosome secretion by tumor cells. Its correlation to anti-PD1/PD-L1 response needs further assessment in the STS population, but has been described in melanoma (97) and lung cancer (98).

There is growing evidence that other immune checkpoints play a role in immune escape in STS. LAG-3 is highly expressed on the surface of both peripheral and tumor-infiltrating T cells of patients with STS (99). Dufresne et al. demonstrated that LAG-3 was expressed across all immune subgroups (76). As with PD-L1, LAG-3 expression is associated with a richer CD8+ infiltrate and a poorer prognosis. Interestingly, CTLA-4, LAG-3 and TIM-3 proteins are often coexpressed with PD-(L)1, which provides a strong rationale for association therapies in STS (84, 100). A study combining nivolumab (anti-PD1) and relatlimab (anti-LAG-3) is currently recruiting (NCT04095208).

In addition, other immunologic checkpoint proteins play a major role in immune escape. In Dufresne et al, ICOS (*Inducible T-cell costimulator*) and GITR (*glucocorticoid-induced tumor necrosis factor receptor*) seemed to be of major interest in all studied histotypes, as was OX40 in synovialosarcoma and CD40 in GIST and synovialosarcoma based on RNA-sequencing data (76). Macrophage-related checkpoints are also of reat interest. CD47 is expressed on tumor cell surface, on a bimodal way (either on 0% or >90% of tumor cells), protecting them from phagocytosis by an interaction with SIRPα on macrophage/dendritic cell surface (61). CD47 and SIRPα expression depend on the tumor histotype with a high frequency of expression in DDLPS and chordoma, as assessed by immunohistochemistry (61). Anti-CD47 antibodies and SIRPα inhibitors have shown promising activities in various cancer types (101–103). CSF1-R is also a commonly expressed target across all immune subgroups (76). Eventually, evidence is growing on the crucial role played by indoleamine-2,3-dioxygenase (IDO) in tumor escape. Overall, IDO protein overexpression is described in 40-60% of STS, which seems correlated to PD-L1 expression and CD8+ infiltrate (86, 104).

### Immune Infiltrate Is Correlated to Survival

Several studies have correlated the immunologic infiltrate with prognosis in sarcoma. For example, tumors with a greater CD8+ T-cell infiltrate, the presence of M1 macrophages, and plasma cells appear to have a better prognosis (80–82, 105, 106). A high density of CD20+ lymphocytes is correlated to CD8+ infiltrate (84) and also seems associated with a better outcome (84, 85). Conversely, a microenvironment rich in M0 and M2 macrophages correlated with a lower CD8+ T-cell infiltrate and a worse prognosis (76, 87).

As suggested in several studies (107–109) and summarized in meta-analyses (110–112), PD-L1 overexpression is associated with a poorer overall survival. In Budczies et al, the worst prognosis was found among patients harboring a PD-L1 copy number gain and a low CD3+ infiltrate (92). This trend is also observed for other immunological checkpoints. For example, SIRPα and CD47 in myxofibrosarcoma, synovialosarcoma and osteosarcoma (61) or LAG3 across all subtypes (99) are associated with a worse prognosis, as assessed by immunohistochemistry. All these observations suggest the importance of immunological checkpoint proteins in tumor escape.

### Tumor Microenvironment Is Associated With Response to PD-1 Blockade in STS

In a correlative analysis of the SARC028 trial assessing Pembrolizumab monotherapy in STS, PD-L1 was only expressed by tumor cells in 2 of 40 tumors with evaluable biopsy (113). Both of them responded to treatment, but the 5 other responders did not express PD-L1. PD-L1 expression by TAMs was also associated with response to Pembrolizumab. Pooling the results of 9 multicenter immunotherapy trials, Italiano et al. analyzed 153 patients who received an anti-PD-1/PD-L1 monotherapy (47). Of 21 PD-L1 positive tumors, 6 patients had an objective response (28.5%), against only 9 of 133 (6.7%) patients in PD-L1 negative tumors. As previously suggested, sPD-L1 expression is of great interest in STS. Its correlation to anti-PD1/PD-L1 has been described in melanoma (97) and lung cancer (98) but has not been assessed in STS, to our knowledge.

Similarly, in the correlative study of SARC028, the immune infiltrate of responders at baseline showed a higher density of CD8+ T cells compared to non-responders (113). As it has been proven in other tumor types (3), baseline CD8+ T cells seem therefore necessary to achieve a response under anti-PD(L)1 treatment.

Strikingly, Petitprez et al. showed that the immune high subclass E, characterized by the presence of TLS, predicted response to PD-1 blockade therapy in a prospective multi- centre phase 2 clinical trial of pembrolizumab in STS (SARC028). This was based on the retrospective analysis of biopsies from 47 patients included in the study and with available tumor material demonstrating the feasibility of the implementation of this biomarker in the routine setting. Tumours were considered TLS-positive when a CD3 aggregate with DC-Lamp staining was found juxtaposing a CD20 aggregate. Only aggregates with surface above 60,000 µm², containing at least 700 cells and at least 350 CD20+ cells were considered. This predictive value of a response to immunotherapy in sarcoma was confirmed at ASCO 2021 with an update of the PembroSarc (pembrolizumab and cyclophosphamide in STS) study. Of the 240 patients included, 48 were TLS+, and in these patients, there was a 26.7% response rate (compared with 2.1% in the others) and a progression-free survival at 6 months of 40% (compared with 4.2% in the others) (114). It has been recently demonstrated in a pan-tumor model that TLS are associated with a better response rate and better survival independently of PD-L1 expression and CD8+ T cell density (115).

Therefore, it seems that a promising approach for ICIs would be trials driven by TME, based on PD-L1 expression and on the presence of TLS and B cells. However, beyond ICIs monotherapy, which are probably efficient in biomarker-selected STS, other new innovative approaches have been designed to increase ICI response.

## Improving Immunotherapy Efficacy: Beyond Immune Checkpoint Inhibitors

### Combinations With ICI Backbone

Before entering details of combination trials, it should be mentioned that STS histotypes have varying sensitivities to chemotherapy and tyrosine kinase inhibitors (TKI). Chemoresistance of clear cell sarcomas and ASPS is fully established, whereas, UPS and synovial sarcoma (116) are more chemosensitive. On the other hand, ASPS (117), solitary fibrous tumors (118) and extraskeletal myxoid chondrosarcoma (119) are known for their sensitivity to TKIs. On the contrary, LPS are known to be resistant to antiangiogenics (120). This should be taken into account when interpreting results of combination trials.

#### Antiangiogenics

There is a strong pre-clinical rationale to combine anti-VEGF therapies with ICIs (121, 122) and this combination has proven its efficacy in other cancer types, notably renal cell carcinoma (123). Angiogenesis and immune infiltrates are strongly correlated in the TME, and immune cells express angiogenic receptors.

Wilky et al. reported on a phase 2 single-arm, single centre trial of axitinib and pembrolizumab association (124). This trial included 33 patients, with an intention-to-treat (ITT) population of 32 patients. The ORR of the ITT population was 25%, the three month PFS rate was 65.6% and median PFS was 4.7 months. Notably, this trial included six LMS, two DDLPS, five UPS and 12 ASPS. Results in the ASPS population were particularly interesting with an ORR of 54.5%, a three months PFS rate of 72.7% and a median PFS of 12.4 months. In the non-ASPS population (N=21), ORR was 9.5%, the three months PFS rate was 61.9% and median PFS was 3 months.

The IMMUNOSARC trial is a phase 1b/2 trial of nivolumab and sunitinib in STS (125). The recommended phase 2 dose was set at 37.5mg daily sunitinib as induction therapy for two weeks followed by 25mg daily sunitinib in combination with nivolumab. As a whole, the ORR was 21%. The six months PFS rate was 48% and median PFS was 5.6 months.

#### Chemotherapy

Regarding immunotherapy combinations with chemotherapy, these are now standard-of-care in non-small-cell lung cancer (12). The hope of these combinations is that induction of immunogenic cell death by chemotherapy could synergize with immunotherapy and render it more effective (126–129). Moreover, combination of chemotherapy prevents hyperprogressive disease which has been reported in some cases of STS (130). Regional hyperthermia is a commonly used treatment in locally advanced sarcomas in combination with neoadjuvant chemotherapy (131). Even if no trial has tried to associate it with ICI to our knowledge, it should be noted that regional hyperthermia and neoadjuvant chemotherapy increase the count of TILs and reduce FoxP3+ infiltration, providing a rationale for their association with ICI (132).

##### Cyclophosphamide

Toulmonde et al. led a phase 2 non-randomized multicentric trial which included 57 patients of which 15 LMS patients, 16 UPS patients, 16 other sarcomas (notably 2 DDLPS) and a group of GIST patients (N=10) (86). This trial tested the association of pembrolizumab 200mg every three weeks with metronomic cyclophosphamide as an immune regulatory drug.

Results of this trial were fairly disappointing. Overall, no response was observed across all subgroups. PFS rate at 6 months was 0% in the LMS group and 0% in the UPS group. In the other STS group, one patient responded to treatment, 8 had disease progression as best response and 5 had stable disease. PFS rate at 6 months was 14.3%. As updated at ASCO 2020, in this cohort, only one patient had TLS present in the TME, as assessed by immunohistochemistry (114).

##### Doxorubicin

Standard-of-care metastatic first line treatment in STS is anthracycline-based chemotherapy. Combination therapies of anthracyclines with other drugs have continuously been disappointing for the past 30 years (25, 26, 31, 133, 134). However, a role of innate and adaptive immunity in anthracycline’s activity has been suggested (135).

*Pollack et al.* report on the first combination trial of doxorubicin and pembrolizumab in an anthracyline-naive population of sarcomas (136). This was a combined phase 1/2 trial studying two doses of doxorubicin (45 and 75mg/m2), in 37 patients. The recommended phase 2 dose was doxorubicin 75mg/m2 for 6 cycles combined with pembrolizumab 200mg flat dose for a total of two years. Nine patients (24%) had already had at least one prior line of systemic treatment. In the combined phase 1/2 trial, seven partial responses were seen for an ORR of 19%, median PFS was 8.1 months, PFS rate at 12 weeks was 81% and median OS was 27.6 months. This trial was closed earlier than expected inclusions because of insufficient efficacy. Notably, two of four DDLPS and two of three UPS exhibited prolonged responses.

Livingstone et al. reported at ASCO 2020 on preliminary results of a pilot study of doxorubicin and pembrolizumab in 30 STS patients with promising results (137). Reported ORR was 36.7% overall, 40% in the LMS group (N=4/10), 28.6% in the LPS group (N=2/7) and 100% in UPS group (N=3/3). Disease control rate was 80% overall with median PFS of 6.9 months and median OS 15 months.

Overall, combination of doxorubicin with ICI has a promising activity but still needs to be thoroughly evaluated and compared to standard-of-care of chemotherapy in a randomized phase III trial. Notably, the choice of the comparator arm will depend on precise setting and histotype: Adriamycin single agent or in combination with ifosfamide or dacarbazine.

##### Trabectedin

Trabectedin is a DNA-binder of marine origin. This cytotoxic drug’s precise efficacy mechanism is still under investigation but an immune-mediated response has been described: trabectedin induces an apoptosis of monocytes and macrophages both in peripheral blood and in the tumor and is associated with reduced angiogenesis (138). Therefore, combination of trabectedin and ICIs has a strong rationale for synergism. Trabectedin monotherapy has been reported to be particularly efficient in LMS, DDLPS and translocation-related sarcomas (139).

The SAINT trial’s preliminary results were presented at ASCO 2020 (140) by Gordon et al. This phase 2 trial investigated the efficacy and safety of the combination of ipilimumab 1mg/kg every 12 weeks with nivolumab 3mg/kg every two weeks and trabectedin 1.2mg/kg every three weeks in 41 STS patients. The combination yielded four complete responses, five partial responses and 27 stabilisation: ORR was 22% and disease control rate 87.9%. As a comparison, in the phase 3 trial of trabectedin solely in LMS and LPS, ORR was 9.9% and disease control rate was 61% (139). In translocation-related sarcomas, ORR is 8% and disease control rate is 65% (141).

The TRAMUNE trial is a phase 1b trial investigating the efficacy of durvalumab and trabectedin in STS and ovarian cancers (142). Nine patients were included in the escalation dose phase and recommended phase 2 dose was trabectedin 1.2mg/m2 with durvalumab 1120mg flat dose every three weeks. The STS expansion cohort consisted of 14 patients. The ORR was 7.1%, the non-progression rate was 64%, the six month PFS rate was 28.6% and the median PFS was 2.3 months.

##### Eribulin

Eribulin is a microtubule-depolymerizing drug. This antimitotic agent is currently recommended in advanced LPS and LMS (143). Its antitumor activity also relies on modulation of the TME *via* tumor vascular remodelling (leading to an increased tumor perfusion but a reduced VEGF expression) and on its effect on epithelial-to-mesenchymal transition (144, 145).

Nathenson et al. reported on the LMS cohort of a phase 2 study of eribulin 1.4mg/m2 on day 1 and day 8 in combination with pembrolizumab on day 1 of every three week cycles (146). Of 19 enrolled patients, two partial responses and five stabilised diseases were reported, for an ORR of 10.5% and non-progression rate of 36.8%. As a comparison, the registration trial of eribulin in LPS and LMS found an ORR of 4% and a 12 week PFS rate of 33% (143).

**Figure 1:**
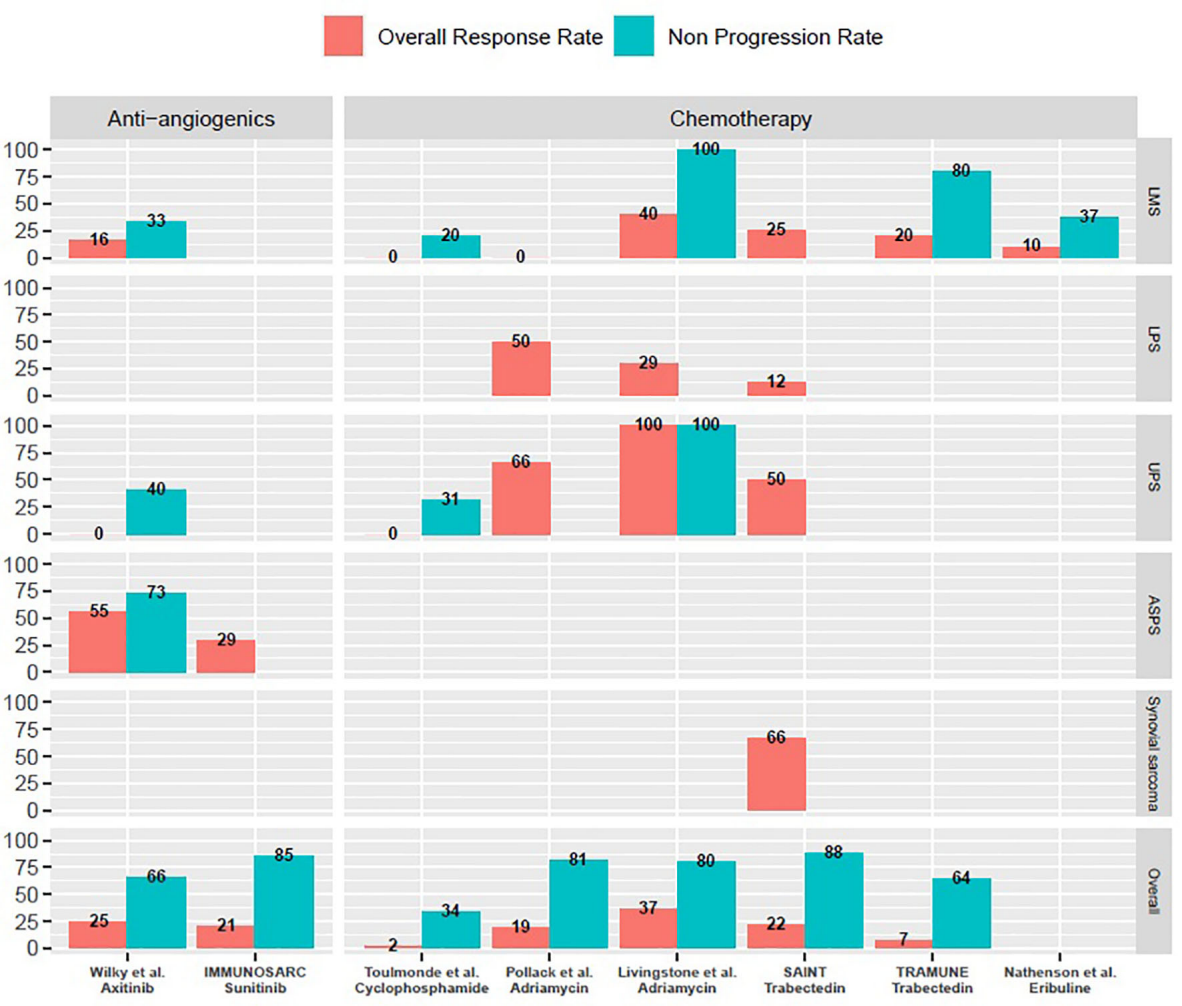
Overall response rates and non-progression rates of selected prospective trials of combination treatments with immune-checkpoint inhibitors mentioned in the manuscript. *ASPS, Alveolar soft-part sarcomas; LMS, Leiomyosarcoma; LPS, Liposarcoma; UPS, Undifferentiated pleomorphic sarcomas. M, months; w, weeks. Only cohorts including a minimum of three patients are reported on this graph. Non-progression rates are either three months progression-free survival rates or disease control rates at first evaluation, as reported in specific trials and mentioned throughout the manuscript*.

As a whole, combination therapies with ICIs are feasible with manageable toxicity profiles. All of them yielded promising activity in specific subtypes (**Figure 1**). More translational data is awaited in order to better select potentially responsive histotypes for future registration trials.

### Other Approaches to Immunotherapy

Numerous other approaches of immunotherapy exist today. Notably: molecules targeting other checkpoints (LAG-3, TIM-3, OX40, TIGIT), modified cytokines, bi-specific antibodies, metabolic targets (IDO-1 and tryptophan, adenosine and CD39/CD73), oncolytic viruses, therapies targeting NK-cells, dendritic cell therapies, vaccines, modified T-cells and chimeric antigen receptor (CAR) T-cell therapies, amongst others.

For the purpose of this review, we will focus on the treatments which have moved forward in clinical trials specifically in the sarcoma field.

#### Oncolytic Viruses

The intra-tumoral approach has gained growing interest in the immunotherapy field as it yields the promise of greater benefit for less (147). Moreover, regarding immune-based cancer treatments, TAAs are the primers and the targets, and are abundantly present in the tumor (148, 149). A variety of intra-tumoral approaches exist and are promising in the sarcoma field, the most advanced is an oncolytic virus approach (150).

Mechanisms of oncolytic viruses are double: direct lysis of tumor cells and induction of an immune response by immunogenic death (151). Other mechanisms of action, notably on the TME, enhance their activity (152). Oncolytic viruses are genetically modified in order to target tumor cells, which can be done by using specific traits of tumor cells such as TERT polymerase activation, RAS signalling or TP53 pathway (153). Toxicity seems acceptable, but their efficacy can be limited by multiple factors: clearance of virus by the immune system or induction of exhaustion on lymphocytes of the TME, for instance.

In line with these limitations, combination therapy with ICIs hold great promise to synergize with oncolytic viruses and potentiate their activity (154–159).

Kelly et al. recently reported an impressive ORR of 35% in 20 patients with STS in the advanced setting treated by the combination of Talimogene laherparepvec (T-VEC) and pembrolizumab (160). T-VEC is a herpes simplex virus type 1 oncolytic virus, injected intratumorally and approved by the US Food and Drug Administration (FDA) for treatment of melanoma (161). The combination of T-VEC intratumorally and pembrolizumab intravenously at 200mg flat dose was administered every 21 days. PFS rate at 12 weeks was 70%, PFS rate at 24 weeks was 39.4% and median PFS was 17.1 weeks.

#### Vaccination

Vaccination against cancer includes a variety of modalities and targets. The general idea is to stimulate the adaptive immune system against TAAs presented to lymphocytes by antigen-presenting cells. The antigens are tumor specific and can either be identified targets with specific cancer neoantigens or whole tumor lysates.

The vaccination method can be immune cell-mediated, using antigen presenting cells such as dendritic cells, peptide based or nucleic acid based (162, 163). As a whole, these methods have shown exciting pre-clinical data and interesting early phase trials with limited activity in monotherapy.

It is likely that these immunotherapies will be further developed in combination with other treatment modalities: chemotherapy, radiotherapy or ICIs, in a prime and boost approach. Most of the clinical research in the STS field has been focused on CTAs.

##### Dendritic Cell Based Immune Therapies

Dendritic cells (DCs) are at the frontier between adaptive and innate immunity. When activated upon danger signals, these cells process and present TAAs to lymphocytes, activating them against these specific TAAs. DC-based vaccination might be able to trigger an immune response in sarcomas.

CDX1401 is a DC targeted antibody linked to NY-ESO-1 peptide, which has been combined with immune stimulating substances in a phase 1 trial of 45 patients including 5 sarcomas (164).This regimen allowed for stimulation of an NY-ESO-1 targeted response with no clinical responses but an interesting disease control rate (N=13/45) which lasted for a median of 6.7 months. Interestingly, eight patients underwent subsequent ICI therapy and six of them responded to this subsequent ICI therapy, suggesting a potential synergy to be exploited in future trials.

The LV305 vaccine is a first-in-class viral vector which is able to transduce specifically DCs and induce the expression and presentation of NY-ESO-1 by MHC class 1. It is injected intradermally, every 3 weeks. The first-in-human trial included 39 patients, 24 sarcomas, namely 13 synovial sarcomas and six MRLPS (165). There were only two partial responses, including one sarcoma. Amongst patients with sarcomas, the 3-month non-progression rate was 62.5% and median PFS was 4.6 months. Correlative studies showed better PFS and OS in patients with pre-treatment specific NY-ESO-1 immune responses and patients with induced specific NY-ESO-1 immune responses.

Following this first trial, a phase 2 trial tested the combination of LV305 with a TLR4 agonist and atezolizumab (anti-PDL1), compared to atezolizumab alone in patients with synovial sarcoma or MRLPS (166). As a whole, the combination displayed a minimal non-significant benefit over atezolizumab alone. However, patients in the combination arm were more heavily pre-treated and were more frequently metastatic rather than relapsing locally. A significant specific immune response was significantly more frequently developed in the combination arm. This combination warrants further assessment in earlier phases of the disease.

##### Peptide Vaccines

Several trials have shown the safety of peptide vaccines on NY-ESO-1 CTA and on SYT-SSX protein fusion of synovial sarcomas. These trials have tested different schedules, doses and forms of peptides. All of these trials have shown that immune and clinical responses can be achieved depending on the type of peptide and the schedule of administration (167–170). These were small phase 1 trials but larger trials, potentially in combination with ICIs are awaited.

#### Modified T-Cell Therapies

##### Modified TCR T-Cells

Modified T-cells therapy are autologous T-cells transduced with a viral vector in order to express a specific T-cell receptor (TCR) for a tumor-specific antigen. Two CTA targets in the sarcoma field have yielded promising response rates: NY-ESO-1 and MAGEA4.

Two trials have reported on different T-cell engineered NY-ESO-1 specific TCR. The first one included 18 patients with synovial sarcoma of advanced stage, with 11 patients experiencing response. The estimated three year OS was 38% (171). The second trial included 12 synovial sarcoma patients and found an ORR of 50% (172). This trial expanded in order to test for other lymphodepleting regimens and efficacy in NY-ESO-1 low expressing patients (173, 174). ORR in the four cohorts of synovial sarcoma patients ranged from 20 to 50%. The highest response rate was in the initial cohort with high dose lymphodepleting regimen and high expression of NY-ESO-1. The IGNYTE-ESO protocol study is currently evaluating this therapy in treatment-naive and previously treated synovial sarcomas.

Van Tine et al. reported on a trial of modified autologous T-cell, transduced to express a HLA*A02 restricted TCR targeting MAGEA4 in 16 synovial sarcomas. Of note, one death was reported due to aplastic anemia secondary to lymphodepleting regimen. The ORR was 44% and the disease control rate was of 94% (175).

##### CAR-T Cells

CAR T-cells have displayed impressive results in hematologic malignancies. Clinical results in solid tumors have been somewhat disappointing up to now. CAR T-cells are different from modified TCR-T cells, as their receptor is not a TCR but a specific antibody derived single chain variable fragment, fused with T-cell signaling domain and co-activators. This means these modified T-cells are not MHC-restricted and are more specific of the target.

In the sarcoma field, most data is pre-clinical and has already been reviewed elsewhere (176). One clinical report of Her2 directed CAR T-cells in osteosarcoma, Ewing sarcoma and desmoplastic small round cell tumor displayed interesting results (177).

## Improving Immunotherapy Efficacy: Histotype-Tailored Immuno-Oncology

Facing the challenge of heterogeneity of clinical course and TME across sarcoma histotypes, as well as the diversity of new molecules available, it seems crucial to design specific clinical trials based on the better understanding of the biology of specific subtypes.

### Leiomyosarcomas

#### Clinical and Biological Background of LMS

Leiomyosarcoma accounts for roughly 10-20% of all STS, and has a crude incidence rate of around 0.7/100.000/y (178). Two distinct subtypes are usually distinguished: uterine LMS (ULMS) are less frequent than soft-tissue LMS (STLMS). The TCGA cohort helped define the molecular biology of these tumors: LMS is globally associated with complex genomics and a high number of CNA (52). On the other side, the mutational burden is low, with a median mutational burden ranging from 1.5 to 2.5mut/Mb, and only 0.7% (STLMS) and 0.9%(ULMS) harbor more than 20mut/Mb (51, 64). The most frequent copy number loss affects chromosome 10 (loss of PTEN) (179), and on the whole, molecular alterations often concern *TP53*, *RB1* as well as *PTEN*. The PIK3CA/AKT pathway is often hyperactivated (180), without a clear difference between STLMS and ULMS. The two subtypes however have distinct methylation profiles and mRNA signatures: ULMS have a higher DNA damage response score, are characterized with homologous recombination deficiency (HRD) in 20% of cases, and with a loss of BRCA2 in 6.5% (181). Moreover, ULMS is known to express hormone receptors in around 40% of cases (182). On the other side, STLMS have a more activated hypoxia-induced factor-1α (HIF-1α) pathway.

On the whole, the sensitivity of LMS towards chemotherapy is moderate, and first-line treatment relies on doxorubicin-based regimen (31) or on gemcitabine-docetaxel association (183). In later setting, options rely on chemotherapy with dacarbazine, trabectedin, eribulin. Antiangiogenic therapies, especially pazopanib, have also demonstrated a meaningful activity in LMS (184). Despite these treatment options, metastatic LMS still has a poor prognosis with an overall survival around 14 months (185).

#### Immune Microenvironment of LMS

As we previously described, LMS is characterized by complex genomics with a high number of CNA, but a lower mutational burden than UPS (64). On the whole, LMS is to be considered as poorly infiltrated by CD8+ T cells (88, 186), even though there is an heterogeneity, with a richly infiltrated subset. TAMs are more represented, although less than in other sarcomas of complex genomics, and M2/M1 ratio is high and correlated to a homologous recombination deficiency, CNAs but also to a poorer prognosis (61, 187). In studies using RNA-sequencing, LMS express PD-L1 (62, 63), LAG-3 and TIM-3 in around 50-60%, 15% and 10% (64) of cases, respectively.

Petitprez et al. analysed 189 LMS in their cohort: most of them were classified in the SIC A (immune desert) and SIC B (immune-low), and only a few tumors harbored TLS, which were described as rather immature and localized in the periphery of the tumor (84).

#### Immunotherapy in LMS: Next Steps

Results of phase II trials have been quite disappointing in advanced LMS so far, with an ORR of 6.9% and a non-progressive rate of 54% (47). These results are corroborated by a phase 2 trial showing no response to anti-PD1 monotherapy (43), and by a better comprehension of the TME of LMS. New approaches are therefore needed to improve the outcome of this disease.

As we described, PTEN is lost in around 60% of LMS (179), and PIK3CA/AKT pathway is therefore often pathologically activated, leading to cell proliferation and to an inhibition of apoptosis. This loss of PTEN was also identified as a resistance mechanism to anti-PD1 therapy in a metastatic LMS (188), which was also found in other tumor types (189). Therefore, it would be interesting to associate PD-1 blockade to PIK3CA/AKT inhibition (PIK3CA or mTOR inhibitors).

It has also been found that IFNγ-induced JAK-STAT pathway activation was responsible for an increase in PD-L1 and IDO-1 expression in metastatic LMS, providing the rationale for an association of PD-1 and IDO blockade (190).

One of the issues in LMS is its poorer CD8+ infiltrate, compared to other complex genomics sarcomas, probably hampering its response to ICI. It would be interesting to increase its antigenicity and radiotherapy could be an interesting option (191). Associating ICI with PARP inhibitors to induce more neoantigens could also be an interesting attitude, especially in ULMS, associated with HRD deficiency. A last strategy would be to associate ICI with chemotherapy: clinical trials associating anti-PD1 to gemcitabine or eribuline are ongoing (*see* **Table 2**).

**Table 2:** Immunological characteristics of the main subtypes of STS.

	Subtype	Crude Incidence Rate	TMB	Molecular alterations		TLS	Immune infiltrate	PD-L1 and other CP(mRNA expression)
LMS	Uterine LMS	0.2/100.000/y	1.5-2.5	Complex genomics: high number of CNAFrequent TP53, RB1, PTEN alterationNo pathognomonic alteration Molecular targets: -PIK3CA/AKT pathway -IGF-1R	Hormone receptor: 40-70%HRD : 20%	Absent	Low CD8+ infiltrate Low TAM infiltrate compared to other SCG More TAMs than TILsHigh M2/M1 ratio	PDL1: 50-60% LAG-3: 15% TIM-3: 10% CD47 low SIRPα low
	Soft tissue LMS	0.51/100.000/y			NA			
LPS	DDLPS	0.81/100.000/y	1.7	12q13-15 amplification MDM2-CDK4 overexpression		Present	**Inflamed tumor** High TIL density More TAMs than TILs FoxP3+ cells present CD20+ cells present	PD-L1: 20% LAG-3: 77% TIM-3: 88% SIRPα +++ CD47 +++
	WDLPS						Lower TIL density than in DDLPS TAMs present	
	MRLPS	0.1/100.000/y		t (12,16) translocation SSX-SS18 fusion protein		NA	**Immune desert** Low TIL density TAMs present: essentially M2, correlated with PI3K pathway	PD-L1<10% SIRPα+++
	Pleomorphic LPS	<5% LPS		Frequent TP53 and RB1 alteration No pathognomonic alteration		Absent	Low TIL density High TAMs/TILs ratio	PD-L1: 0% SIRPα +++ CD47 +++
UPS	Two molecular subtypes: - immune high - FGFR	0.46/100.000/y	2.5-5	Complex genomics: high number of mutations and CNA Frequent TP53, RB1 and ATRX alterations No pathognomonic alteration Molecular targets: -PI3K/AKT pathway -RAS/MAPK pathway		Present	**Inflamed tumor** High TIL infiltrate Clonal TCR repertoire High TAMs infiltrate More TAMs than TILs: M2>M1 Important FoxP3 contingentCD20+ cells present	PD-L1 30-50% LAG3 72% TIM3 83% SIRPα in ~30%CD47 low
Translocation-associated sarcomas	Synovial sarcoma	0.13/100.000/y	1.7	t(X;18)(p11.2;q11.2) translocation SS18:SSX fusion protein Frequent EZH2 overexpression, TP53 alteration and MDM2 amplification		Absent	**Immune desert** Low TIL density Low TAM infiltrate	PD-L1<10% 60% TIM-3 30% LAG-3 SIRPα low CD47 low
	ASPS	<0.1/1.000.000/y	NA	t(X;17)(p11;q25), ASPSCR1-TFE3		NA	Low TIL density High M2/M1 ratio	SIRPα in ~30%CD47 low

SCG, Sarcomas of complex genomics, TAM, tumor-associated macrophage; TIL, tumor-infiltrating lymphocyte; TLS, Tertiary lymphoid structures; CNA, Copy-number alteration; ASPS, Alveolar soft-part sarcomas; LMS, Leiomyosarcoma; WDLPS, well-differentiated liposarcoma; DDLPS, dedifferentiated liposarcoma; MRLPS, myxoid round-cell liposarcoma; pLPS, pleomorphic liposarcoma; UPS, Undifferentiated pleomorphic sarcomas; NA, not applicable.

### Liposarcomas

#### Clinical and Biological Background of Liposarcomas

Liposarcomas have an incidence around 0.8-1/100.000/year. This group of sarcomas is composed of three distinct entities: well-differentiated and dedifferentiated LPS (WDLPS and DDLPS), MRLPS and pleomorphic LPS (192, 193).

WDLPS and DDLPS represent 50% of LPS, the primitive tumor localization is retroperitoneal in 45% of cases and they are characterized molecularly by the supernumerary ring chromosome and/or giant marker chromosome of amplified 12q13-15 region, leading to the amplification of MDM2, CDK4 and HMGA2 genes (194). These LPS are less chemosensitive than other LPS (195), although combination chemotherapy with anthracycline remains the most active regimen (196, 197). WDLPS have a more indolent course, tend to recur locally and in 10% of cases have a DDLPS component.

MRLPS account for 40% of LPS, they are characterized in >95% of cases by a chromosomal translocation involving chromosomes 12 and 16, leading to the fusion protein FUS-DDIT3. Importantly, 95% of MRLPS express NY-ESO-1 CTA (71) in immunohistochemistry. Myxoid LPS is the more indolent counterpart of round cell LPS, which are defined by having more than 5% of small round cells in a myxoid LPS. MRLPS are radiosensitive (198) and more chemosensitive than DDLPS/WDLPS (195), with a particular responsiveness regarding trabectedin (199).

Pleomorphic LPS account for 5% of LPS, are more aggressive and poorly characterized.

#### Immune Microenvironment of LPS

As a whole, LPS seem to be infiltrated by T-cells and express PDL1 (63, 186). In regards to separate histotypes of LPS, a general pattern seems to emerge: DDLPS are more infiltrated and express more PDL1, whereas MRLPS seem to be less infiltrated, and WDLPS are probably somewhere in the middle (62, 100, 107, 200), though the latter has frequently been grouped with DDLPS in reported studies.

DDLPS and WDLPS seem to have an expression of roughly 10-20% PDL1 (62, 94, 108, 200, 201), both in immunohistochemistry and gene expression studies. Compared to other STS, DDLPS seem to be in the higher proportions of TILs and PDL1 expression, though consistently reporting less infiltration than certain subtypes such as UPS (62, 100, 108, 202). TILs are present in over 50% of cases (61, 108, 200) with presence of NK cells, B-cells, TLS and expression of co-inhibitory checkpoints such as LAG3 and TIM3 (100, 201, 203). However, T-cell infiltration seems to be less oligoclonal than LMS or UPS (62). Regarding TAMs, DDLPS are amongst the STS containing the most TAMs, but with an important infiltration by T-cells so the ratio TAM/TILs is in the average, and close to the WDLPS ratio (61).

MRLPS are poorly infiltrated by T-cells, have a low PDL1, low TCR clonality and are infiltrated by TAMs with a majority of M2 type macrophages (62, 100). PDL1 expression is usually less than 10% (94, 200). The importance of macrophages and their prognostic value has been reported specifically in MRLPS (204). Interestingly, 18.5% of MRLPS are mutated for the PI3KCA gene which correlates with the absence of TILs (200) and the presence macrophages (204). These tumors are amongst those with the highest ratio of M2/M1 TAMs and expressing the most SIRPα (61).

Regarding pleomorphic LPS, data is scarce and inconsistent. As a whole, these tumors have markers of bad prognosis, with little to no T-cell infiltration (94, 200), absence of TLS (201) and high TAMs and M2 infiltration (61).

#### Immunotherapy in LPS: Next Steps

As discussed previously, results of ICI in LPS have been promising at first, with strong rationale as these tumors are infiltrated and express PDL1. However, further assessment has been disappointing with an ORR of roughly 8% (47).

DDLPS and WDLPS seem to have some degree of response to ICIs, but monotherapy is insufficient. Interesting results have been reported in combination with chemotherapy, as displayed in **Figure 1** (136, 137). Another perspective could be combinations with molecularly targeted therapies, namely MDM2 antagonists and anti-CDK4/6. MDM2 antagonists have achieved interesting results as monotherapy in DDLPS, though with important myelotoxicity (205). Importantly, MDM2 had been suggested as a mechanism of hyperprogression in ICIs (206), making it a good target for combination with ICIs. Moreover, interesting preclinical data shows antagonizing MDM2 enhances T-cell killing (207) and NK-cell mediated killing (208) with a synergism when combined to antiPD1 (209). Likewise, CDK4/6 inhibitors have shown limited responses in LPS (210) but trigger antitumor immunity (211), which could potentially synergize with ICIs. Epigenetic modifications are crucial in LPS (212) and synergism between epigenetic drugs and immunotherapy has a strong rationale (213). Finally, combination with other immune-based therapies could be efficient, notably other ICIs, as DDLPS are the STS type with the highest expression of LAG3 and TIM3 (100).

As discussed previously, MRLPS have high expression of NY-ESO-1 CTA, low TILs and low clonality of TCR. Results of modified TCR T-cell based immunotherapy have been promising, and this seems to be an efficient therapy, which is being tested in earlier phases of the disease. Another interesting option to pursue, might be combination with trabectedin, as this drug is immunogenic (138, 214) and particularly efficient in MRLPS (199). Likewise, radiotherapy combinations with ICIs hold great promise (215): radiotherapy increases PDL1 expression in STS (93) and MRLPS are particularly sensitive to radiotherapy (198). Moreover, combination of radiotherapy and trabectedin seems interesting in MRLPS (216), and might be combined as a whole with ICIs.

Regarding pleomorphic LPS, more translational and clinical data for this specific histotype is needed in order to better define its sensitivity to immunotherapy.

### Undifferentiated Pleomorphic Sarcomas

#### Clinical and Biological Background of UPS

Undifferentiated pleomorphic sarcomas used to be called malignant fibrous histiocytoma (MFH). These sarcomas are characterized by their absence of distinct features of differentiation (217–219).

UPS/MFH was the most frequent type of STS but as molecular phenotyping techniques have progressed, it seems that misdiagnosis was a frequent problem and differentiation of certain phenotypes was identified within certain misclassified UPS/MFH (220–223). However, UPS remains one of the three most frequent histotypes of STS with LPS and LMS (52) and one of the worst prognosis (224).

UPS are complex genomic sarcomas, have the highest CNA and mutation rate with frequent alterations of TP53 and RB1 (52, 221). Multiple studies have tried to better characterize their molecular background (225–231). Of note, some studies have suggested that there is a continuum between MFS and UPS (52) whereas others have found LMS and UPS to be molecularly similar (232–235). Investigations report on different molecular alterations with different potential prognostic (230–232, 234, 236–238) or therapeutic implication (221). Other studies have shed light on subsets of UPS with alterations on the PI3K/Akt pathway (225, 237), the Hippo pathway (52, 234) or RAS/MAPK pathway (225, 230, 237) with particular attention to FGF (226, 227). In particular, recently a multi-omics approach has separated UPS in two molecular subtypes: one immune-high and one expressing FGFR (227), which is consistent with previous data (229). As a whole, UPS is probably a heterogeneous histotype, with distinct molecular subtypes which might benefit from more extensive molecular testing before treatment (220).

#### Immune Microenvironment of UPS

Looking at tumor genome, UPS have amongst the highest TMB in sarcomas, with angiosarcomas (51, 225). In line with this higher mutation rate, T-cell infiltrates in UPS have a more oligoclonal TCR repertoire than other sarcomas (62).

As a whole, UPS are infiltrated by TILs (64), with notably presence of CD8+ and FoxP3+ T-cells (91, 100, 239) and TLS. TILs express immune checkpoint proteins, though less than in DDLPS, with 36% of cases with PD1+ TILs, 72% of cases with LAG3+ TILs and 83% TIM3+ TILs (100) by immunohistochemistry. PDL1 is highly expressed, roughly 30-50% of cases (94, 106).

TAMs are predominant, with a clear majority of M2 phenotype. In fact, UPS are the second histotype most infiltrated by TAMs, after angiosarcomas, and share the highest M2 proportion with LMS. Notably, UPS have a low expression of CD47 and moderate SIRPa expression on TAMs (61).

This histotype microenvironment seems to be more immune infiltrated than other STS (62). However, as previously mentioned, this seems to be particularly true for a subset of UPS (227).

#### Immunotherapy in UPS: Next Steps

Up to now, clinical data of ICI in UPS have reported ORRs of 15-40% with ICI alone and over 50% when combining ICIs with other molecules (47), amongst the highest. For UPS, the next step is probably better selection of patients with biomarkers for personalized medicine. In the immune-high subset of UPS, ICIs might be particularly boosted by combination with radiotherapy, which has proven to increase PDL1 expression in STS (93), and particularly UPS (36, 240).

Notably, UPS are particularly responsive to combination of ICIs (41). For other molecular subtypes, driven by particular targets, combination ICI with FGFR inhibitors might be beneficial (241) and should be further explored. Combination of ICI with oncolytic virus is also of great interest in this histotype: in the phase 2 trial evaluating Pembrolizumab with T-VEC, the 2 patients with UPS treated with this regimen had a partial response.

### Translocation-Related Sarcomas: Synovial Sarcoma and Alveolar Soft-Part Sarcoma

#### Clinical and Biological Background of Translocation-Related Sarcomas

Translocation-related sarcomas are driven by a chromosomal translocation, leading to the expression of a fusion protein. For clarity reason, we chose to focus only on synovial sarcomas (the most frequent subtype) and ASPS (with an impressive response rate to ICI).

Synovial sarcoma accounts for nearly 10% of all STS and can occur in any anatomic site, preferentially in adults younger than 30 years old (242). It is characterized by the presence of a pathognomonic translocation between chromosome X and chromosome 18, t(X,18)(p11.2;q11.2) leading to the expression of an SS18:SSX fusion protein. This SS18:SSX protein binds and to the BAF (*BRG1-associated factors*) complex and activates it, thus displacing the tumor suppressor INI1 (coded by the gene *SMARCB1*). This epigenetic phenomenon is eventually responsible for a repression of E-cadherin, a downregulation of BCl2, and a downregulation of MCL1 (243–245). Another consequence is the pathological expression of CTAs, such as NY-ESO-1 (*see supra*). These epigenetic mechanisms in synovial sarcoma can be emphasized by an overexpression of EZH2 (*enhancer of zeste homologue 2*), which is described in poorly differentiated synovial sarcomas, and seems associated with a poorer prognosis (246).

Despite a relatively good comprehension of its biology, the prognosis of synovial sarcoma remains poor, although chemosensitive. In the metastatic setting, treatment relies on anthracycline-based chemotherapy, but another option is high-dose ifosfamide alone, to which synovial sarcoma is known to be sensitive (247). Pazopanib and antiangiogenic therapies are also particularly interesting in this particular histotype (184, 248).

ASPS is a much rarer sarcoma subtype, accounting for less than 1% of all STS and occurring preferentially in young adults as well (249). Even though around 55% are metastatic at diagnosis, they tend to have a more indolent course than other histological subtypes, with a 5-year overall survival of 61% in case of a metastatic disease (250). It is characterized by the t(X;17)(p11;q25) translocation, which codes a chimeric ASPSCR1-TFE3 transcription factor, and is indirectly responsible for an overexpression of genes related to angiogenesis (among which c-Met or VEGF), cell proliferation and metastasis (251). ASPS is known for its sensitivity to TKI therapy with a response rate around 30% (252, 253), counterbalancing a poor response rate to anthracycline chemotherapy (254).

#### Immune Microenvironment of Translocation-Related Sarcomas

Translocation-related sarcomas are supposedly less immunogenic, due to a poor immunogenicity of fusion proteins and a lower mutational burden. Synovial sarcoma is no exception to this with a median TMB around 1.7mut/Mb (51) and only 1% of tumors harboring more than 20mut/Mb. However, they are characterized by a high CTA expression: as we previously described, 49-76% of synovial sarcomas express NY-ESO-1 and 51% of them coexpress NY-ESO-1 and MAGE-A4 and PRAME (72–74).

Translocation-related sarcomas have a poorer CD3+ infiltrate than non translocation-related sarcomas and 21% of them show no TIL (100). Concerning synovial sarcomas, this immune desert is also associated with a low TCR clonality and a lower expression of antigen presentation genes (62). They also have one of the lowest TAM infiltrate (61) across all STS subtypes. They are associated with a resting mast cell and a naive B cell signature. Moreover, immune-checkpoint proteins are rarely expressed in translocation-associated sarcomas: 64% are described as PD-1, LAG-3 and TIM-3 negative in immunohistochemistry (100). In synovial sarcoma, there is no PD-L1 expression by tumor cells, and only 20% of them have PD-L1 expressing TAMs in immunohistochemistry (88). In Petitprez et al., SIC E synovial sarcomas were a minority (20%), whereas SIC A and B (immune deserts) represented around 50% (84).

Data regarding TME of ASPS is scarce due to the tumor rarity. They are described as poorly infiltrated as well, with a low CD3+, CD8+ and FoxP3+ infiltrate (100). Among translocation-associated sarcomas, they are however described as having the highest CD163+ expression, responsible for one of the highest M2/M1+M0 ratios (61).

#### Immunotherapy in Translocation-Related Sarcomas: Next Steps

Given the overexpression of CTA, synovial sarcoma was initially considered as a good candidate for ICI. The first trial used anti-CTLA-4 monotherapy with ipilimumab in 6 patients, but no response was observed (42). Overall, ICIs have failed to demonstrate a meaningful clinical benefit in synovial sarcoma, with a response rate under 10%, if no response at all (*see* **Table 1** and **Figure 1**). This is probably the result of the global immune desert of synovial sarcomas. However, if epigenetic mechanisms play a major role in the oncogenesis of synovial sarcomas, the synergy of epigenetic drugs with ICI should be assessed in this tumor type, for example with an association of EZH2 inhibitors and ICI in tumors overexpressing EZH2.

With the association of specific neoantigens (NY-ESO-1 and MAGE-A4) and the absence of immunosuppressive microenvironment, the future of immunotherapy in synovial sarcoma probably lies in the targeting of specific neoantigens. Adoptive cellular therapies targeting NY-ESO-1 (171) have shown impressive results, so did engineered autologous T-cells targeting MAGE-A4 (175). Vaccine therapies, such as LV305 have also shown interesting results (165) and are currently assessed in several clinical trials (*see* **Table 2**). It should be noted that ICI could be of interest in combination with these therapies, as suggested by the stunning response rate of ICI after failure of CDX1401 treatment (164).

On the other hand, ASPS is probably the histotype with the most impressive benefit from ICI with a global ORR of 48.8% for anti-PD(L)1 monotherapy (47). The reason for this sensitivity remains unclear in a histotype characterized by a poor immune infiltrate.

Given its known sensitivity to TKI such as pazopanib and the known synergy between these treatments, the association of ICI and antiangiogenics needs further assessment, but results are impressing so far with the association of Axitinib and Pembrolizumab harboring an ORR of 54.5%, a three months PFS rate of 72.7% and median PFS was 12.4 months (124).

## Discussion

Immunotherapy and ICIs have raised great promises in the oncology field, and are now standard-of-care in a large variety of cancers. Unfortunately, these treatments have demonstrated a more moderate clinical benefit in STS with an ORR estimated around 10-15% for anti-PD(L)1 monotherapy. These results have yet to be considered as encouraging, in a disease where treatments beyond the first-line harbor a modest ORR (10% for trabectedin (139) and 4% for eribulin (143) in L-sarcomas, 6% for Pazopanib in non-adipocytic STS), and where the ultimate goal is often disease stabilization. Moreover, data is accumulating on the benefit of an early introduction of immunotherapeutic agents (87, 255), and ICIs should therefore be assessed earlier in the course of STS. In this context, neoadjuvant trials are ongoing and will be of particular interest (*see* **Table 3**).

**Table 3:** Ongoing clinical trials of immunotherapy in STS.

NCT	Histotype	Setting	Molecule	Phase	Number of patients	Estimated Completion Date
**Immune checkpoint inhibitors**						
NCT03141684	ASPS	Metastatic	Atezolizumab	II	46	October 2021
NCT02815995	All STS	Metastatic	Durvalumab + tremelimumab	II	150	August 2020
NCT04274023	Clear Cell Sarcoma	Metastatic	TSR-042 (anti-PD1)	II	16	May 2024
NCT04480502	UPS	Metastatic	Envafolimab (anti-PD-L1) +/- ipilimumab	II	160	July 2022
NCT04118166	All STS	Metastatic	Nivolumab + ipilimumab + cryotherapy	II	30	October 2025
NCT03465592	All STS, <40yo	Metastatic	Nivolumab following relapse after allogeneic bone marrow transplant	II	39	March 2026
NCT03465592	MPNST	Neoadjuvant	Nivolumab + ipilimumab	II	18	January 2025
NCT02691026	MPNST	Metastatic	Pembrolizumab	II	18	December 2025
**Antiangiogenic therapy + Immune checkpoint inhibitors**						
NCT03711279	All STS	Metastatic	Camrelizumab (anti-PD1) + apatinib vs. doxorubicin + ifosfamide	II	289	September 2022
NCT04551430	All STS	Metastatic	Cabozantinib + nivolumab + ipilimumab	II	105	January 2027
NCT03798106	All STS	Metastatic	Pazopanib + durvalumab	II	37	August 2022
NCT03277924	All STS	Metastatic	Nivolumab + sunitinib	I/II	270	September 2022
NCT04172805	All STS	Metastatic	Anlotinib + toripalimab (anti-PD1)	II	70	June 2022
NCT03946943	UPS	Metastatic	Anlotinib + toripalimab (anti-PD1)	II	25	July 2023
**Other targeted therapies + Immune checkpoint inhibitors**						
NCT04438824	WDLPS, DDLPS	Metastatic	Palbociclib + INCMGA00012 (anti-PD1)	II	30	June 2023
NCT04216953	All STS	Metastatic	Cobimetinib + atezolizumab	I/II	120	February 2024
NCT04624178	LMS	Metastatic	Rucaparib + nivolumab	II	20	November 2022
NCT03126591	All STS	Metastatic	Olaratumab + pembrolizumab	I	41	March 2021
**Chemotherapy + Immune checkpoint inhibitors**						
NCT03899805	LPS, LMS, UPS	Metastatic	Eribulin + pembrolizumab	II	57	August 2024
NCT03802071	All STS	Metastatic, anthracycline-naïve	Doxorubicin + durvalumab	II	44	August 2022
NCT03317457	All STS	Metastatic, anthracycline-naïve	Doxorubicin + durvalumab + tremelimumab	II	100	June 2022
NCT04535713	All STS	Metastatic	Gemcitabine + doxorubicine + docetaxel + nivolumab	II	260	September 2025
NCT03719430	All STS	Metastatic	Doxorubicin + APX005M (CD40 agonistic antibody)	II	27	December 2023
NCT04356872	UPS, Synovial Sarcoma, DDLPS, MRLPS	Metastatic, anthracycline-naïve	Sintilimab (anti-PD1) + doxorubicin + ifosfamide	II	45	March 2023
NCT04606108	All STS	Neoadjuvant	Camrelizumab + neoadjuvant chemotherapy (investigator’s choice)	II	63	March 2024
NCT04577014	All STS	Metastatic	INCMGA00023 (anti-PD1) + gemcitabine + docetaxel	II	74	September 2022
NCT04028063	All STS	Metastatic, anthracycline-naïve	Doxorubicin + AGEN1884 (anti-CTLA-4) + AGEN2034 (anti-PD1)	II	28	November 2022
NCT03512834	Angiosarcoma	Metastatic, first line	Paclitaxel + avelumab	II	32	May 2023
NCT03536780	LMS	Metastatic, second line	Gemcitabine + avelumab	II	38	February 2022
NCT03123276	All STS	Metastatic	Gemcitabine + pembrolizumab	I/II	24	December 2020
NCT03085225	All STS	Metastatic	Trabectedin + durvalumab	Ib	50	May 2021
NCT03590210	All STS	Metastatic	Trabectedin + nivolumab	II	92	July 2022
NCT03138161	All STS	Metastatic	Trabectedin + nivolumab + ipilimumab	I/II	45	March 2021
NCT04650984	All STS	Metastatic, anthracycline-naïve	Doxorubicin vs. doxorubicin + L19TNF (TNFα agonist)	III	102	December 2024
NCT04332874	UPS, ASPS	Metastatic	Isolated limb infusion (dactinomycin + melphalan) + pembrolizumab	II	30	April 2023
**Epigenetic therapy + Immune checkpoint inhibitors**						
NCT04025931	All STS	Metastatic	Chidamide (HDAC inhibitor) + toripalimab (anti-PD1)	II	53	July 2021
**Radiotherapy + Immune checkpoint inhibitors**						
NCT03116529	All STS	Neoadjuvant	Durvalumab + tremelimumab + radiotherapy	II	35	June 2022
NCT03463408	All STS	Neoadjuvant	Nivolumab + ipilimumab + radiotherapy	II	24	August 2025
NCT03307616	UPS DDLPS	Neoadjuvant	Nivolumab +/- ipilimumab	II	32	October 2021
NCT03548428	LMS, UPS, LPS	Metastatic	Stereotactic ablative radiotherapy + atezolizumab	II	103	December 2021
NCT02992912	All STS	Metastatic	Stereotactic ablative radiotherapy + atezolizumab	II	187	October 2020
NCT03338959	All STS	Metastatic	Radiation therapy + pembrolizumab	II	26	June 2022
NCT03092323	UPS, WDLPS, DDLPS	Neoadjuvant + adjuvant	Radiotherapy vs. radiotherapy + pembrolizumab	II	102	July 2025
NCT03474094	All STS	Neoadjuvant + adjuvant	Radiotherapy + atezolizumab	II	22	July 2021
**New immunotherapeutic agents + Immune checkpoint inhibitors**						
NCT04668300	Angiosarcoma DDLPS Osteosarcoma	Metastatic	Durvalumab + oleclumab (anti-CD73)	II	75	June 2024
NCT04095208	All STS	Metastatic	Relatlimab (anti-LAG3) + nivolumab	II	67	September 2024
NCT03063632	Synovial Sarcoma	Metastatic	IFNγ-1b + pembrolizumab	II	30	May 2021
NCT04242238	All STS	Metastatic	Avelumab + DCC-3014 (anti-CSF1R)	Ib	48	January 2022
NCT03282344	Chondrosarcoma, Osteosarcoma, UPS, vascular sarcoma, ASPS, DDLPS, pLPS, LMS	Metastatic	Nivolumab + NKTR-214 (pegylated IL-2)	II	85	April 2021
NCT04420975	All STS	Metastatic	Nivolumab + intratumoral BO-112 (double-strand RNA)	I	25	January 2025
NCT03414229	All STS	Metastatic	Pembrolizumab + epacadostat	II	30	January 2021
**Immune checkpoint inhibitors + Oncolytic virus**						
NCT03069378	All STS	Metastatic	T-VEC + pembrolizumab	II	60	March 2021
NCT03886311	All STS	Metastatic	T-VEC + nivolumab + trabectedin	II	40	December 2022
**Vaccine therapy**						
NCT01883518	All STS	Metastatic	Autologous DC vaccine loaded with allogeneic tumor lysate expression of CTA	II	48	September 2020
NCT01803152	All STS	Metastatic	Autologous DC vaccine loaded with allogeneic tumor lysate expression of CTA + gemcitabine	I	19	July 2024
NCT02700230	MPNST	Metastatic	Intratumoral administration of measles virus genetically engineered to express NF1	I	30	June 2021
**Adoptive cellular therapy**						
NCT03725605	All STS	Metastatic	Intratumoral LTX-315 (oncolytic peptide) followed by TIL culture, expansion and infusion	II	6	February 2023
NCT04044768	HLA-A*02, MAGE-A4 expressing MRLPS/Synovial Sarcoma	Metastatic	Anti-MAGE-A4 SPEAR T-cell	II	45	November 2034
NCT02650986	Tumors expressing NY-ESO1*	Metastatic	NY-ESO1 TCR-transduced TILs +/- decitabine	I/IIa	27	June 2021
NCT04052334	All STS, <40yo	Metastatic	TIL infusion + IL-2 after lymphodepletion	I	15	August 2022
NCT03250325	Synovial Sarcoma with HLA-A*02:01 or HLA-A*02:06	Metastatic	NY-ESO-1-specific TCR gene transduced T cells	I/II	8	January 2020
NCT03399448	Synovial Sarcoma MRLPS	Metastatic	NY-ESO-1-specific TCR gene transduced T cells	I	NA	October 2020
NCT03240861	HLA-A*0201 positive, NY-ESO-1 expressing tumors*	Metastatic	NY-ESO-1-specific TCR gene transduced PBMC + PBSC	I	12	September 2022
NCT03450122	HLA-A*0201 positive, NY-ESO-1 expressing STS	Metastatic	NY-ESO-1-specific TCR gene transduced T cells + IL-2 +/- DC-targeting lentiviral vector LV305 or CMB305 (LV305 + NY-ESO-1 protein vaccine + TLR4)	II	18	June 2021
NCT00902044	HER2-positive sarcoma	Metastatic	HER-2-CAR T cells	II	36	July 2032
NCT03356782	STS with at least 1 target antigen positive IHC	Metastatic	Sarcoma-specific CAR T cells	I/II	20	December 2023
NCT04318964	HLA-A*0201 positive, NY-ESO-1 expressing STS	Metastatic	TAEST16001 cells : NY-ESO-1-specific TCR gene transduced T cells + IL-2	I	12	June 2022

ASPS, Alveolar soft-part sarcomas; LMS, Leiomyosarcoma; WDLPS, well-differentiated liposarcoma; DDLPS, dedifferentiated liposarcoma; MRLPS, myxoid round-cell liposarcoma; pLPS, pleomorphic liposarcoma; UPS, Undifferentiated pleomorphic sarcomas; DC, dendritic cell; TCR, T-cell receptor; TLR4, toll-like receptor 4; CAR, chimeric antigen receptor; TNFα, Tumor Necrosis Factor α; HDAC, histone deacetylase; CSF1R, colony stimulating factor 1 receptor; PBMC, peripheral blood myeloid cells; PBSC, peripheral blood stem cells; TIL, tumor infiltrating lymphocyte.

Clinical trials are currently ongoing, evaluating association therapies, especially with chemotherapy and anti-PD-(L)1 combination. Preliminary results of such associations have proven hopeful, in particular the association of Doxorubicin and anti-PD1 therapy, with an interesting overall response rate of 37% (137) in an early setting. Oncolytic viruses are also of great interest in STS, and yield an impressive ORR of 35% with the association of T-VEC and Pembrolizumab.

Even if histotype is an important aspect of tumor heterogeneity in STS, there are still large variations in TME inside STS subtypes and responses to ICI have been described in nearly every type. The model of complex and simple genomics sarcomas shows several limitations: as previously mentioned, LMS, the most frequent complex genomics STS, is probably one of the STS subtypes with the most disappointing response rates, while responses to ICI have been described in translocation-associated sarcomas, particularly ASPS which displays the most promising ORR.

**Figure 2:**
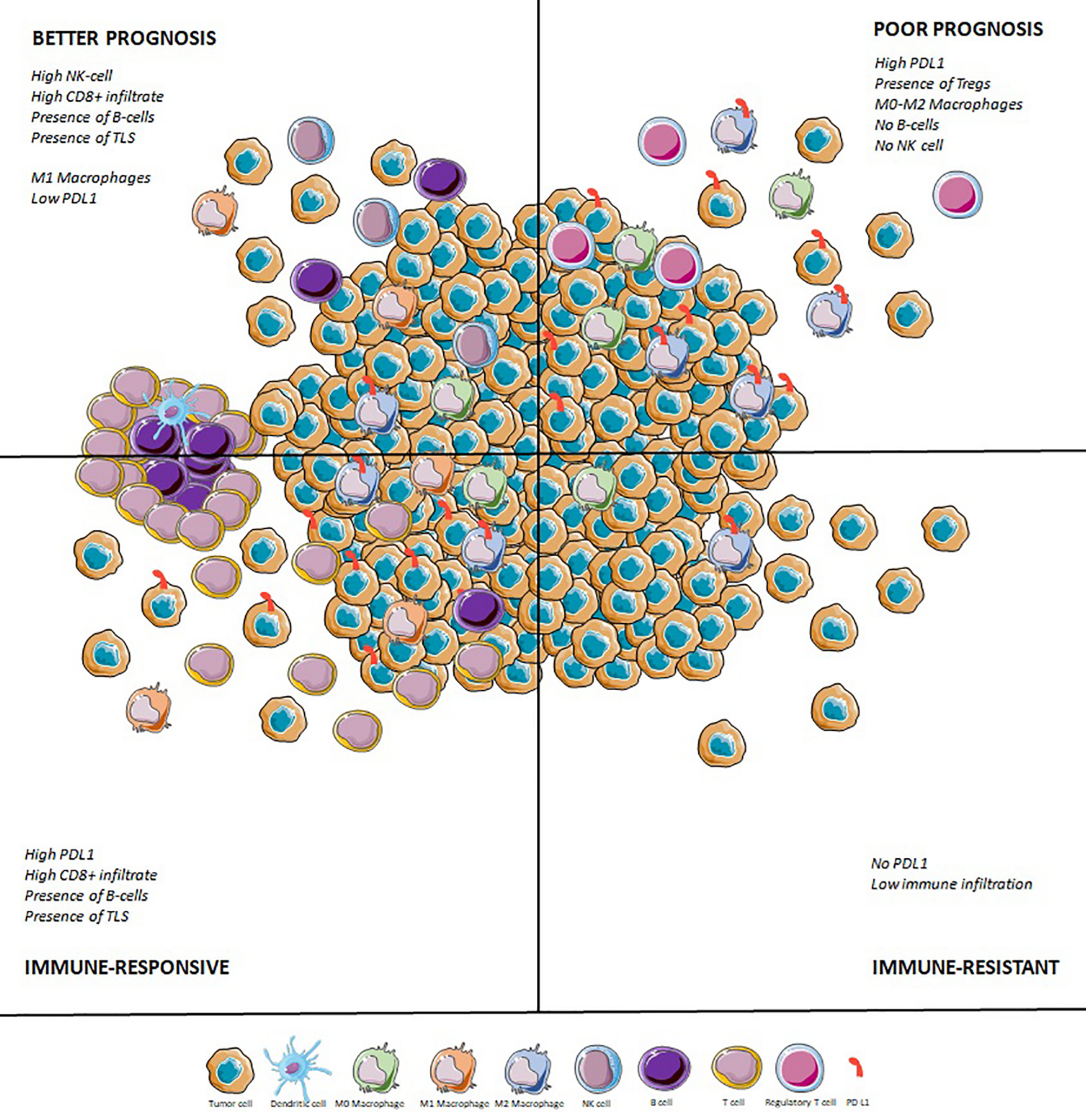
Immune tumor microenvironment in sarcomas: prognostic and predictive impact. *TLS, Tertiary lymphoid structures; TMB, Tumor mutational burden*.

Eventually, it seems there will not be a one-size-fits-all immunotherapy in STS: immuno-oncology development in the field will require careful selection of the right immune-based therapy for the right histotype and the right TME. Patients with STS should be included in immune-checkpoint inhibitor trials based on their histotype (UPS, DDLPS) and on their TME [PD-L1 and other ICP expression, CD8+ T cell infiltrate, presence of TLS, (**Figure 2**)]. Some histotypes are characterized by an overexpression of CTA, especially synovial sarcomas or MRLPS. For these patients, immunotherapy perspectives should rely on peptide vaccine or adoptive cellular therapies.

As we have tried to suggest in this review, the path forward for immunotherapy in sarcomas might be by integrating biomarkers and translational data in the very early phases of drug development.

## Author Contributions

MRD and EN contributed equally to conception and design of the review and wrote the first draft of the manuscript. AI and RB provided a careful reviewing and wrote sections of the manuscript. All authors contributed to manuscript revision, read, and approved the submitted version.

## Conflict of Interest

The authors declare that the research was conducted in the absence of any commercial or financial relationships that could be construed as a potential conflict of interest.

## Publisher’s Note

All claims expressed in this article are solely those of the authors and do not necessarily represent those of their affiliated organizations, or those of the publisher, the editors and the reviewers. Any product that may be evaluated in this article, or claim that may be made by its manufacturer, is not guaranteed or endorsed by the publisher.

## References

[1] Galon J Bruni D. Tumor Immunology and Tumor Evolution: Intertwined Histories. Immunity (2020) 52:55–81. doi: 10.1016/j.immuni.2019.12.018 31940273

[2] Waldman AD Fritz JM. Lenardo MJ. A Guide to Cancer Immunotherapy: From T Cell Basic Science to Clinical Practice. Nat Rev Immunol (2020) 20:651–68. doi: 10.1038/s41577-020-0306-5 PMC7238960 32433532

[3] Tumeh PC Harview CL Yearley JH Shintaku IP Taylor EJM Robert L. PD-1 Blockade Induces Responses by Inhibiting Adaptive Immune Resistance. Nature (2014) 515:568–71. doi: 10.1038/nature13954 PMC4246418 25428505

[4] Iwai Y Ishida M Tanaka Y Okazaki T Honjo T Minato N. Involvement of PD-L1 on Tumor Cells in the Escape From Host Immune System and Tumor Immunotherapy by PD-L1 Blockade. Proc Natl Acad Sci (2002) 99:12293–7. doi: 10.1073/pnas.192461099 PMC129438 12218188

[5] Leach DR Krummel MF Allison JP. Enhancement of Antitumor Immunity by CTLA-4 Blockade. Science (1996) 271:1734–6. doi: 10.1126/science.271.5256.1734 8596936

[6] McCarthy EF. The Toxins of William B. Coley and the Treatment of Bone and Soft-Tissue Sarcomas. Iowa Orthop J (2006) 26:154–8. PMC1888599 16789469

[7] Chen DS Mellman I. Elements of Cancer Immunity and the Cancer-Immune Set Point. Nature (2017) 541:321–30. doi: 10.1038/nature21349 28102259

[8] Schreiber RD Old LJ Smyth MJ. Cancer Immunoediting: Integrating Immunity’s Roles in Cancer Suppression and Promotion. Science (2011) 331:1565–70. doi: 10.1126/science.1203486 21436444

[9] Hodi FS O’Day SJ McDermott DF Weber RW Sosman JA Haanen JB. Improved Survival With Ipilimumab in Patients With Metastatic Melanoma. N Engl J Med (2010) 363:711–23. doi: 10.1056/NEJMoa1003466 PMC3549297 20525992

[10] Larkin J Chiarion-Sileni V Gonzalez R Grob J-J Rutkowski P Lao CD. Five-Year Survival With Combined Nivolumab and Ipilimumab in Advanced Melanoma. N Engl J Med (2019) 381:1535–46. doi: 10.1056/NEJMoa1910836 31562797

[11] Reck M Rodríguez-Abreu D Robinson AG Hui R Csőszi T Fülöp A. Pembrolizumab Versus Chemotherapy for PD-L1-Positive Non-Small-Cell Lung Cancer. N Engl J Med (2016) 375:1823–33. doi: 10.1056/NEJMoa1606774 27718847

[12] Gandhi L Rodríguez-Abreu D Gadgeel S Esteban E Felip E De Angelis F. Pembrolizumab Plus Chemotherapy in Metastatic Non-Small-Cell Lung Cancer. N Engl J Med (2018) 378:2078–92. doi: 10.1056/NEJMoa1801005 29658856

[13] Powles T Kockx M Rodriguez-Vida A Duran I Crabb SJ van der Heijden MS. Clinical Efficacy and Biomarker Analysis of Neoadjuvant Atezolizumab in Operable Urothelial Carcinoma in the ABACUS Trial. Nat Med (2019) 25:1706–14. doi: 10.1038/s41591-019-0628-7 31686036

[14] Motzer RJ Escudier B McDermott DF George S Hammers HJ Srinivas S. Nivolumab Versus Everolimus in Advanced Renal-Cell Carcinoma. N Engl J Med (2015) 373:1803–13. doi: 10.1056/NEJMoa1510665 PMC5719487 26406148

[15] Casali PG. Soft Tissue and Visceral Sarcomas: ESMO–EURACAN Clinical Practice Guidelines for Diagnosis, Treatment and Follow-Up. Ann Oncol (2018) 29:17. doi: 10.1093/annonc/mdy096 29846498

[16] Stiller CA Botta L Brewster DH Ho VKY Frezza AM Whelan J. EUROCARE-5 Working Group. Survival of Adults With Cancers of Bone or Soft Tissue in Europe-Report From the EUROCARE-5 Study. Cancer Epidemiol (2018) 56:146–53. doi: 10.1016/j.canep.2018.08.010 30179828

[17] CDM F JA B PCW H F M. WHO Classification of Tumours of Soft Tissue and Bone. Available at: https://publications.iarc.fr/Book-And-Report-Series/Who-Classification-Of-Tumours/WHO-Classification-Of-Tumours-Of-Soft-Tissue-And-Bone-2013 (Accessed January 25, 2021).

[18] Burningham Z Hashibe M Spector L Schiffman JD. The Epidemiology of Sarcoma. Clin Sarcoma Res (2012) 2:14. doi: 10.1186/2045-3329-2-14 23036164 PMC3564705

[19] Casali PG. Gastrointestinal Stromal Tumours: ESMO–EURACAN Clinical Practice Guidelines for Diagnosis, Treatment and Follow-Up. Ann Oncol (2018) 29:11. 30188977 10.1093/annonc/mdy320

[20] Italiano A Mathoulin-Pelissier S Cesne AL Terrier P Bonvalot S Collin F. Trends in Survival for Patients With Metastatic Soft-Tissue Sarcoma. Cancer (2011) 117:1049–54. doi: 10.1002/cncr.25538 20945333

[21] Savina M Le Cesne A Blay J-Y Ray-Coquard I Mir O Toulmonde M. Patterns of Care and Outcomes of Patients With METAstatic Soft Tissue SARComa in a Real-Life Setting: The METASARC Observational Study. BMC Med (2017) 15. doi: 10.1186/s12916-017-0831-7 PMC5385590 28391775

[22] Le Cesne A. Making the Best of Available Options for Optimal Sarcoma Treatment. Oncology (2018) 95(Suppl 1):11–20. doi: 10.1159/000494861 30554197

[23] Blay J-Y Honoré C Stoeckle E Meeus P Jafari M Gouin F. Surgery in Reference Centers Improves Survival of Sarcoma Patients: A Nationwide Study. Ann Oncol (2019) 30:1143–53. doi: 10.1093/annonc/mdz124 PMC6637376 31081028

[24] Blay J-Y Soibinet P Penel N Bompas E Duffaud F Stoeckle E. Improved Survival Using Specialized Multidisciplinary Board in Sarcoma Patients. Ann Oncol (2017) 28:2852–9. doi: 10.1093/annonc/mdx484 PMC5834019 29117335

[25] Benjamin RS Wiernik PH Bachur NR. Adriamycin Chemotherapy–Efficacy, Safety, and Pharmacologic Basis of an Intermittent Single High-Dosage Schedule. Cancer (1974) 33:19–27. doi: 10.1002/1097-0142(197401)33:1<19::aid-cncr2820330107>3.0.co;2-m 4810094

[26] Tap WD Wagner AJ Schöffski P Martin-Broto J Krarup-Hansen A Ganjoo KN. Effect of Doxorubicin Plus Olaratumab vs Doxorubicin Plus Placebo on Survival in Patients With Advanced Soft Tissue Sarcomas: The ANNOUNCE Randomized Clinical Trial. JAMA (2020) 323:1266–76. doi: 10.1001/jama.2020.1707 PMC7139275 32259228

[27] Tap WD Jones RL Van Tine BA Chmielowski B Elias AD Adkins D. Olaratumab and Doxorubicin Versus Doxorubicin Alone for Treatment of Soft-Tissue Sarcoma: An Open-Label Phase 1b and Randomised Phase 2 Trial. Lancet Lond Engl (2016) 388:488–97. doi: 10.1016/S0140-6736(16)30587-6 PMC5647653 27291997

[28] Mehnert JM Monjazeb AM Beerthuijzen JMT Collyar D Rubinstein L Harris LN. The Challenge for Development of Valuable Immuno-Oncology Biomarkers. Clin Cancer Res (2017) 23:4970–9. doi: 10.1158/1078-0432.CCR-16-3063 PMC5657536 28864725

[29] Topalian SL Taube JM Anders RA Pardoll DM. Mechanism-Driven Biomarkers to Guide Immune Checkpoint Blockade in Cancer Therapy. Nat Rev Cancer (2016) 16:275–87. doi: 10.1038/nrc.2016.36 PMC5381938 27079802

[30] Veenstra R Kostine M Cleton-Jansen A-M de Miranda NF Bovée JV. Immune Checkpoint Inhibitors in Sarcomas: In Quest of Predictive Biomarkers. Lab Investig J Tech Methods Pathol (2018) 98:41–50. doi: 10.1038/labinvest.2017.128 29155424

[31] Judson I Verweij J Gelderblom H Hartmann JT Schöffski P Blay J-Y. Doxorubicin Alone Versus Intensified Doxorubicin Plus Ifosfamide for First-Line Treatment of Advanced or Metastatic Soft-Tissue Sarcoma: A Randomised Controlled Phase 3 Trial. Lancet Oncol (2014) 15:415–23. doi: 10.1016/S1470-2045(14)70063-4 24618336

[32] Van Glabbeke M Verweij J Judson I Nielsen OS. EORTC Soft Tissue and Bone Sarcoma Group. Progression-Free Rate as the Principal End-Point for Phase II Trials in Soft-Tissue Sarcomas. Eur J Cancer Oxf Engl 1990 (2002) 38:543–9. doi: 10.1016/s0959-8049(01)00398-7 11872347

[33] Borcoman E Nandikolla A Long G Goel S Le Tourneau C. Patterns of Response and Progression to Immunotherapy. Am Soc Clin Oncol Educ Book Am Soc Clin Oncol Annu Meet (2018) 38:169–78. doi: 10.1200/EDBK_200643 30231380

[34] Bernard-Tessier A Baldini C Castanon E Martin P Champiat S Hollebecque A. Patterns of Progression in Patients Treated for Immuno-Oncology Antibodies Combination. Cancer Immunol Immunother CII (2020) 70:221–32. doi: 10.1007/s00262-020-02647-z PMC10973750 32700090

[35] Tazdait M Mezquita L Lahmar J Ferrara R Bidault F Ammari S. Patterns of Responses in Metastatic NSCLC During PD-1 or PDL-1 Inhibitor Therapy: Comparison of RECIST 1.1, irRECIST and iRECIST Criteria. Eur J Cancer Oxf Engl 1990 (2018) 88:38–47. doi: 10.1016/j.ejca.2017.10.017 29182990

[36] Roland CL Keung EZ-Y Lazar AJ Torres KE Wang W-L Guadagnolo A. Preliminary Results of a Phase II Study of Neoadjuvant Checkpoint Blockade for Surgically Resectable Undifferentiated Pleomorphic Sarcoma (UPS) and Dedifferentiated Liposarcoma (DDLPS). J Clin Oncol (2020) 38:11505–5. doi: 10.1200/JCO.2020.38.15_suppl.11505

[37] Neoadjuvant Atezolizumab and Chemotherapy in Patients With Resectable non-Small-Cell Lung Cancer: An Open-Label, Multicentre, Single-Arm, Phase 2 Trial. Lancet Oncol (2020) 21:786–95. doi: 10.1016/S1470-2045(20)30140-6 32386568

[38] Tawbi HA Burgess M Bolejack V Van Tine BA Schuetze SM Hu J. Pembrolizumab in Advanced Soft-Tissue Sarcoma and Bone Sarcoma (SARC028): A Multicentre, Two-Cohort, Single-Arm, Open-Label, Phase 2 Trial. Lancet Oncol (2017) 18:1493–501. doi: 10.1016/S1470-2045(17)30624-1 PMC7939029 28988646

[39] Burgess MA Bolejack V Schuetze S Van Tine BA Attia S Riedel RF. Clinical Activity of Pembrolizumab (P) in Undifferentiated Pleomorphic Sarcoma (UPS) and Dedifferentiated/Pleomorphic Liposarcoma (LPS): Final Results of SARC028 Expansion Cohorts. JCO (2019) 37:11015–11015. doi: 10.1200/JCO.2019.37.15_suppl.11015

[40] D’Angelo SP Mahoney MR Van Tine BA Atkins J Milhem MM Jahagirdar BN. Nivolumab With or Without Ipilimumab Treatment for Metastatic Sarcoma (Alliance A091401): Two Open-Label, non-Comparative, Randomised, Phase 2 Trials. Lancet Oncol (2018) 19:416–26. doi: 10.1016/S1470-2045(18)30006-8 PMC6126546 29370992

[41] Chen JL Mahoney MR George S Antonescu CR Liebner DA Van Tine BA. A Multicenter Phase II Study of Nivolumab +/- Ipilimumab for Patients With Metastatic Sarcoma (Alliance A091401): Results of Expansion Cohorts. J Clin Oncol (2020) 38:11511–1. doi: 10.1200/JCO.2020.38.15_suppl.11511

[42] Maki RG Jungbluth AA Gnjatic S Schwartz GK D’Adamo DR Keohan ML. A Pilot Study of Anti-CTLA4 Antibody Ipilimumab in Patients With Synovial Sarcoma. Sarcoma (2013) 2013:168145. doi: 10.1155/2013/168145 23554566 PMC3608267

[43] Ben-Ami E Barysauskas CM Solomon S Tahlil K Malley R Hohos M. Immunotherapy With Single Agent Nivolumab for Advanced Leiomyosarcoma of the Uterus: Results of a Phase 2 Study. Cancer (2017) 123:3285–90. doi: 10.1002/cncr.30738 PMC5762200 28440953

[44] Blay J-Y Chevret S Penel N Bertucci F Bompas E Saada-Bouzid E. 1619o High Clinical Benefit Rates of Single Agent Pembrolizumab in Selected Rare Sarcoma Histotypes: First Results of the AcSé Pembrolizumab Study. Ann Oncol (2020) 31:S972. doi: 10.1016/j.annonc.2020.08.1845

[45] Kawai A. Efficacy and Safety of Nivolumab Monotherapy in Patients with Unresectable Clear Cell Sarcoma and Alveolar Soft Part Sarcoma (Oscar Trial, Ncch1510): A Multicenter, Phase 2 Clinical Trial; CTOS: Vancouver, BC, Canada, 2020. 10.1002/cncr.35483 39077795

[46] Shi Y Cai Q Jiang Y Huang G Bi M Wang B. Activity and Safety of Geptanolimab (GB226) for Patients With Unresectable, Recurrent, or Metastatic Alveolar Soft Part Sarcoma: A Phase II, Single-Arm Study. Clin Cancer Res (2020) 26:6445–52. doi: 10.1158/1078-0432.CCR-20-2819 33046518

[47] Italiano A Bellera C D’Angelo S. PD1/PD-L1 Targeting in Advanced Soft-Tissue Sarcomas: A Pooled Analysis of Phase II Trials. J Hematol OncolJ Hematol Oncol (2020) 13:55. doi: 10.1186/s13045-020-00891-5 32430039 PMC7236113

[48] Roulleaux Dugage M Jones RL Trent J Champiat S Dumont S. Beyond the Driver Mutation: Immunotherapies in Gastrointestinal Stromal Tumors. Front Immunol (2021) 12:715727. doi: 10.3389/fimmu.2021.715727 34489967 PMC8417712

[49] Samstein RM Lee C-H Shoushtari AN Hellmann MD Shen R Janjigian YY. Tumor Mutational Load Predicts Survival After Immunotherapy Across Multiple Cancer Types. Nat Genet (2019) 51:202–6. doi: 10.1038/s41588-018-0312-8 PMC6365097 30643254

[50] Marabelle A Fakih M Lopez J Shah M Shapira-Frommer R Nakagawa K. Association of Tumour Mutational Burden With Outcomes in Patients With Advanced Solid Tumours Treated With Pembrolizumab: Prospective Biomarker Analysis of the Multicohort, Open-Label, Phase 2 KEYNOTE-158 Study. Lancet Oncol (2020) 21:1353–65. doi: 10.1016/S1470-2045(20)30445-9 32919526

[51] Chalmers ZR Connelly CF Fabrizio D Gay L Ali SM Ennis R. Analysis of 100,000 Human Cancer Genomes Reveals the Landscape of Tumor Mutational Burden. Genome Med (2017) 9. doi: 10.1186/s13073-017-0424-2 PMC5395719 28420421

[52] Cancer Genome Atlas Research Network. Electronic Address: Elizabeth.Demicco@Sinaihealthsystem.Ca, Cancer Genome Atlas Research Network. Comprehensive and Integrated Genomic Characterization of Adult Soft Tissue Sarcomas. Cell (2017) 171:950–65.e28. doi: 10.1016/j.cell.2017.10.014 29100075 PMC5693358

[53] Xu L-B Zhao Z-G Xu S-F Zhang X-X Liu T Jing C-Y. The Landscape of Gene Mutations and Clinical Significance of Tumor Mutation Burden in Patients With Soft Tissue Sarcoma Who Underwent Surgical Resection and Received Conventional Adjuvant Therapy. Int J Biol Markers (2020) 35:14–22. doi: 10.1177/1724600820925095 32520634

[54] Davoli T Xu AW Mengwasser KE Sack LM Yoon JC Park PJ. Cumulative Haploinsufficiency and TriPLoSensitivity Drive Aneuploidy Patterns and Shape the Cancer Genome. Cell (2013) 155:948–62. doi: 10.1016/j.cell.2013.10.011 PMC3891052 24183448

[55] Rosenthal R Cadieux EL Salgado R Bakir MA Moore DA Hiley CT. Neoantigen-Directed Immune Escape in Lung Cancer Evolution. Nature (2019) 567:479–85. doi: 10.1038/s41586-019-1032-7 PMC6954100 30894752

[56] Doyle LA Nowak JA Nathenson MJ Thornton K Wagner AJ Johnson JM. Characteristics of Mismatch Repair Deficiency in Sarcomas. Mod Pathol (2019) 32:977–87. doi: 10.1038/s41379-019-0202-3 30765880

[57] Bonneville R Krook MA Kautto EA Miya J Wing MR Chen H-Z. Landscape of Microsatellite Instability Across 39 Cancer Types. JCO Precis Oncol (2017) 2017. doi: 10.1200/PO.17.00073 PMC5972025 29850653

[58] Campanella NC Penna V Ribeiro G Abrahão-Machado LF Scapulatempo-Neto C Reis RM. Absence of Microsatellite Instability In Soft Tissue Sarcomas. Pathobiol J Immunopathol Mol Cell Biol (2015) 82:36–42. doi: 10.1159/000369906 25766089

[59] Cote GM He J Choy E. Next-Generation Sequencing for Patients With Sarcoma: A Single Center Experience. Oncologist (2018) 23:234–42. doi: 10.1634/theoncologist.2017-0290 PMC5813739 28860410

[60] Lim J Poulin NM Nielsen TO. New Strategies in Sarcoma: Linking Genomic and Immunotherapy Approaches to Molecular Subtype. Clin Cancer Res (2015) 21:4753–9. doi: 10.1158/1078-0432.CCR-15-0831 26330427

[61] Dancsok AR Gao D Lee AF Steigen SE Blay J-Y Thomas DM. Tumor-Associated Macrophages and Macrophage-Related Immune Checkpoint Expression in Sarcomas. Oncoimmunology (2020) 9:1747340. doi: 10.1080/2162402X.2020.1747340 32313727 PMC7153829

[62] Pollack SM He Q Yearley JH Emerson R Vignali M Zhang Y. T-Cell Infiltration and Clonality Correlate With Programmed Cell Death Protein 1 and Programmed Death-Ligand 1 Expression in Patients With Soft Tissue Sarcomas. Cancer (2017) 123:3291–304. doi: 10.1002/cncr.30726 PMC5568958 28463396

[63] Bertucci F Finetti P Perrot D Leroux A Collin F Le Cesne A. PDL1 Expression is a Poor-Prognosis Factor in Soft-Tissue Sarcomas. Oncoimmunology (2017) 6:e1278100. doi: 10.1080/2162402X.2016.1278100 28405501 PMC5384364

[64] Klaver Y Rijnders M Oostvogels A Wijers R Smid M Grünhagen D. Differential Quantities of Immune Checkpoint-Expressing CD8 T Cells in Soft Tissue Sarcoma Subtypes. J Immunother Cancer (2020) 8. doi: 10.1136/jitc-2019-000271 PMC7430493 32792357

[65] Worley BS van den Broeke LT Goletz TJ Pendleton CD Daschbach EM Thomas EK. Antigenicity of Fusion Proteins From Sarcoma-Associated Chromosomal Translocations. Cancer Res (2001) 61:6868–75. 11559563

[66] Baldauf MC Gerke JS Kirschner A Blaeschke F Effenberger M Schober K. Systematic Identification of Cancer-Specific MHC-Binding Peptides With RAVEN. Oncoimmunology (2018) 7:e1481558. doi: 10.1080/2162402X.2018.1481558 30228952 PMC6140548

[67] Pender A Jones RL Pollack S. Optimising Cancer Vaccine Design in Sarcoma. Cancers (2018) 11. doi: 10.3390/cancers11010001 PMC6356514 30577459

[68] Ayyoub M Taub RN Keohan M-L Hesdorffer M Metthez G Memeo L. The Frequent Expression of Cancer/Testis Antigens Provides Opportunities for Immunotherapeutic Targeting of Sarcoma. Cancer Immun (2004) 4:7. 15298487

[69] Wei R Dean DC Thanindratarn P Hornicek FJ Guo W Duan Z. Cancer Testis Antigens in Sarcoma: Expression, Function and Immunotherapeutic Application. Cancer Lett (2020) 479:54–60. doi: 10.1016/j.canlet.2019.10.024 31634526

[70] Kakimoto T Matsumine A Kageyama S Asanuma K Matsubara T Nakamura T. Immunohistochemical Expression and Clinicopathological Assessment of the Cancer Testis Antigens NY-ESO-1 and MAGE-A4 in High-Grade Soft-Tissue Sarcoma. Oncol Lett (2019) 17:3937–43. doi: 10.3892/ol.2019.10044 PMC6403520 30881511

[71] Pollack SM Jungbluth AA Hoch BL Farrar EA Bleakley M Schneider DJ. NY-ESO-1 is a Ubiquitous Immunotherapeutic Target Antigen for Patients With Myxoid/Round Cell Liposarcoma. Cancer (2012) 118:4564–70. doi: 10.1002/cncr.27446 PMC3361576 22359263

[72] Endo M de Graaff MA Ingram DR Lim S Lev DC Briaire-de Bruijn IH. NY-ESO-1 (CTAG1B) Expression in Mesenchymal Tumors. Mod Pathol (2015) 28:587–95. doi: 10.1038/modpathol.2014.155 25412843

[73] Lai J-P Robbins PF Raffeld M Aung PP Tsokos M Rosenberg SA. NY-ESO-1 Expression in Synovial Sarcoma and Other Mesenchymal Tumors: Significance for NY-ESO-1-Based Targeted Therapy and Differential Diagnosis. Mod Pathol (2012) 25:854–8. doi: 10.1038/modpathol.2012.31 PMC6309776 22388761

[74] Iura K Maekawa A Kohashi K Ishii T Bekki H Otsuka H. Cancer-Testis Antigen Expression in Synovial Sarcoma: NY-ESO-1, PRAME, MAGEA4, and MAGEA1. Hum Pathol (2017) 61:130–9. doi: 10.1016/j.humpath.2016.12.006 27993576

[75] Conley AP Wang W-L Livingston JA Ravi V Tsai J-W Ali A. MAGE-A3 is a Clinically Relevant Target in Undifferentiated Pleomorphic Sarcoma/Myxofibrosarcoma. Cancers (2019) 11. doi: 10.3390/cancers11050677 PMC6562561 31096717

[76] Dufresne A Lesluyes T Ménétrier-Caux C Brahmi M Darbo E Toulmonde M. Specific Immune Landscapes and Immune Checkpoint Expressions in Histotypes and Molecular Subtypes of Sarcoma. Oncoimmunology (2020) 9:1792036. doi: 10.1080/2162402X.2020.1792036 32923153 PMC7458655

[77] van Erp AEM Versleijen-Jonkers YMH Hillebrandt-Roeffen MHS van Houdt L Gorris MAJ van Dam LS. Expression and Clinical Association of Programmed Cell Death-1, Programmed Death-Ligand-1 and CD8+ Lymphocytes in Primary Sarcomas is Subtype Dependent. Oncotarget (2017) 8:71371–84. doi: 10.18632/oncotarget.19071 PMC5642642 29050367

[78] Simon M Mughal SS Horak P Uhrig S Buchloh J Aybey B. Deconvolution of Sarcoma Methylomes Reveals Varying Degrees of Immune Cell Infiltrates With Association to Genomic Aberrations. J Transl Med (2021) 19:204. doi: 10.1186/s12967-021-02858-7 33980253 PMC8117561

[79] Tamura R Tanaka T Yamamoto Y Akasaki Y Sasaki H. Dual Role of Macrophage in Tumor Immunity. Immunotherapy (2018) 10:899–909. doi: 10.2217/imt-2018-0006 30073897

[80] Hu C Chen B Huang Z Liu C Ye L Wang C. Comprehensive Profiling of Immune-Related Genes in Soft Tissue Sarcoma Patients. J Transl Med (2020) 18:337. doi: 10.1186/s12967-020-02512-8 32873319 PMC7465445

[81] Deng J Zeng W Kong W Shi Y Mou X. The Study of Sarcoma Microenvironment Heterogeneity Associated With Prognosis Based on an Immunogenomic Landscape Analysis. Front Bioeng Biotechnol (2020) 8:1003. doi: 10.3389/fbioe.2020.01003 32974322 PMC7471631

[82] Gu H-Y Lin L-L Zhang C Yang M Zhong H-C Wei R-X. The Potential of Five Immune-Related Prognostic Genes to Predict Survival and Response to Immune Checkpoint Inhibitors for Soft Tissue Sarcomas Based on Multi-Omic Study. Front Oncol (2020) 10:1317. doi: 10.3389/fonc.2020.01317 32850416 PMC7396489

[83] Morvan MG Lanier LL. NK Cells and Cancer: You can Teach Innate Cells New Tricks. Nat Rev Cancer (2016) 16:7–19. doi: 10.1038/nrc.2015.5 26694935

[84] Petitprez F de Reyniès A Keung EZ Chen TW-W Sun C-M Calderaro J. B Cells are Associated With Survival and Immunotherapy Response in Sarcoma. Nature (2020) 577:556–60. doi: 10.1038/s41586-019-1906-8 31942077

[85] Tsagozis P Augsten M Zhang Y Li T Hesla A Bergh J. An Immunosuppressive Macrophage Profile Attenuates the Prognostic Impact of CD20-Positive B Cells in Human Soft Tissue Sarcoma. Cancer Immunol Immunother CII (2019) 68:927–36. doi: 10.1007/s00262-019-02322-y PMC6529392 30879106

[86] Toulmonde M Penel N Adam J Chevreau C Blay J-Y Le Cesne A. Use of PD-1 Targeting, Macrophage Infiltration, and IDO Pathway Activation in Sarcomas: A Phase 2 Clinical Trial. JAMA Oncol (2018) 4:93. doi: 10.1001/jamaoncol.2017.1617 28662235 PMC5833654

[87] Zhu N Hou J. Assessing Immune Infiltration and the Tumor Microenvironment for the Diagnosis and Prognosis of Sarcoma. Cancer Cell Int (2020) 20:577. doi: 10.1186/s12935-020-01672-3 33292275 PMC7709254

[88] D’Angelo SP Shoushtari AN Agaram NP Kuk D Qin L-X Carvajal RD. Prevalence of Tumor-Infiltrating Lymphocytes and PD-L1 Expression in the Soft Tissue Sarcoma Microenvironment. Hum Pathol (2015) 46:357–65. doi: 10.1016/j.humpath.2014.11.001 PMC5505649 25540867

[89] Sorbye SW Kilvaer T Valkov A Donnem T Smeland E Al-Shibli K. Prognostic Impact of Lymphocytes in Soft Tissue Sarcomas. PLoS One (2011) 6:e14611. doi: 10.1371/journal.pone.0014611 21298041 PMC3029277

[90] Patel SP Kurzrock R. PD-L1 Expression as a Predictive Biomarker in Cancer Immunotherapy. Mol Cancer Ther (2015) 14:847–56. doi: 10.1158/1535-7163.MCT-14-0983 25695955

[91] Que Y Xiao W Guan Y-X Liang Y Yan S-M Chen H-Y. PD-L1 Expression Is Associated With FOXP3+ Regulatory T-Cell Infiltration of Soft Tissue Sarcoma and Poor Patient Prognosis. J Cancer (2017) 8:2018–25. doi: 10.7150/jca.18683 PMC5559963 28819402

[92] Budczies J Mechtersheimer G Denkert C Klauschen F Mughal SS Chudasama P. PD-L1 (CD274) Copy Number Gain, Expression, and Immune Cell Infiltration as Candidate Predictors for Response to Immune Checkpoint Inhibitors in Soft-Tissue Sarcoma. Oncoimmunology (2017) 6:e1279777. doi: 10.1080/2162402X.2017.1279777 28405504 PMC5384369

[93] Patel KR Martinez A Stahl JM Logan SJ Perricone AJ Ferris MJ. Increase in PD-L1 Expression After Pre-Operative Radiotherapy for Soft Tissue Sarcoma. Oncoimmunology (2018) 7:e1442168. doi: 10.1080/2162402X.2018.1442168 29900051 PMC5993497

[94] Vargas AC Maclean FM Sioson L Tran D Bonar F Mahar A. Prevalence of PD-L1 Expression in Matched Recurrent and/or Metastatic Sarcoma Samples and in a Range of Selected Sarcomas Subtypes. PLoS One (2020) 15:e0222551. doi: 10.1371/journal.pone.0222551 32294103 PMC7159201

[95] Zheng B Wang J Cai W Lao I Shi Y Luo X. Changes in the Tumor Immune Microenvironment in Resected Recurrent Soft Tissue Sarcomas. Ann Transl Med (2019) 7:387. doi: 10.21037/atm.2019.07.43 31555701 PMC6736819

[96] Asanuma K Nakamura T Hayashi A Okamoto T Iino T Asanuma Y. Soluble Programmed Death-Ligand 1 Rather Than PD-L1 on Tumor Cells Effectively Predicts Metastasis and Prognosis in Soft Tissue Sarcomas. Sci Rep (2020) 10:9077. doi: 10.1038/s41598-020-65895-0 32493964 PMC7270095

[97] Zhou J Mahoney KM Giobbie-Hurder A Zhao F Lee S Liao X. Soluble PD-L1 as a Biomarker in Malignant Melanoma Treated With Checkpoint Blockade. Cancer Immunol Res (2017) 5:480–92. doi: 10.1158/2326-6066.CIR-16-0329 PMC5642913 28522460

[98] Abu Hejleh T Furqan M Ballas Z Clamon G. The Clinical Significance of Soluble PD-1 and PD-L1 in Lung Cancer. Crit Rev Oncol Hematol (2019) 143:148–52. doi: 10.1016/j.critrevonc.2019.08.009 31675543

[99] Que Y Fang Z Guan Y Xiao W Xu B Zhao J. LAG-3 Expression on Tumor-Infiltrating T Cells in Soft Tissue Sarcoma Correlates With Poor Survival. Cancer Biol Med (2019) 16:331–40. doi: 10.20892/j.issn.2095-3941.2018.0306 PMC6713642 31516753

[100] Dancsok AR Setsu N Gao D Blay J-Y Thomas D Maki RG. Expression of Lymphocyte Immunoregulatory Biomarkers in Bone and Soft-Tissue Sarcomas. Mod Pathol (2019) 32:1772–85. doi: 10.1038/s41379-019-0312-y 31263176

[101] Liu J Wang L Zhao F Tseng S Narayanan C Shura L. Pre-Clinical Development of a Humanized Anti-CD47 Antibody With Anti-Cancer Therapeutic Potential. PLoS One (2015) 10:e0137345. doi: 10.1371/journal.pone.0137345 26390038 PMC4577081

[102] Advani R Flinn I Popplewell L Forero A Bartlett NL Ghosh N. CD47 Blockade by Hu5F9-G4 and Rituximab in Non-Hodgkin’s Lymphoma. N Engl J Med (2018) 379:1711–21. doi: 10.1056/NEJMoa1807315 PMC8058634 30380386

[103] Sikic BI Lakhani N Patnaik A Shah SA Chandana SR Rasco D. First-In-Human, First-In-Class Phase I Trial of the Anti-CD47 Antibody Hu5F9-G4 in Patients With Advanced Cancers. J Clin Oncol (2019) 37:946–53. doi: 10.1200/JCO.18.02018 PMC7186585 30811285

[104] Nafia I Toulmonde M Bortolotto D Chaibi A Bodet D Rey C. IDO Targeting in Sarcoma: Biological and Clinical Implications. Front Immunol (2020) 11:274. doi: 10.3389/fimmu.2020.00274 32194552 PMC7066301

[105] Judge SJ Darrow MA Thorpe SW Gingrich AA O’Donnell EF Bellini AR. Analysis of Tumor-Infiltrating NK and T Cells Highlights IL-15 Stimulation and TIGIT Blockade as a Combination Immunotherapy Strategy for Soft Tissue Sarcomas. J Immunother Cancer (2020) 8:e001355. doi: 10.1136/jitc-2020-001355 33158916 PMC7651745

[106] Boxberg M Steiger K Lenze U Rechl H von Eisenhart-Rothe R Wörtler K. PD-L1 and PD-1 and Characterization of Tumor-Infiltrating Lymphocytes in High Grade Sarcomas of Soft Tissue - Prognostic Implications and Rationale for Immunotherapy. Oncoimmunology (2018) 7:e1389366. doi: 10.1080/2162402X.2017.1389366 29399389 PMC5790346

[107] Kim JR Moon YJ Kwon KS Bae JS Wagle S Kim KM. Tumor Infiltrating PD1-Positive Lymphocytes and the Expression of PD-L1 Predict Poor Prognosis of Soft Tissue Sarcomas. PLoS One (2013) 8:e82870. doi: 10.1371/journal.pone.0082870 24349382 PMC3859621

[108] Orth MF Buecklein VL Kampmann E Subklewe M Noessner E Cidre-Aranaz F. A Comparative View on the Expression Patterns of PD-L1 and PD-1 in Soft Tissue Sarcomas. Cancer Immunol Immunother CII (2020) 69:1353–62. doi: 10.1007/s00262-020-02552-5 PMC11027659 32222780

[109] Kim C Kim EK Jung H Chon HJ Han JW Shin K-H. Prognostic Implications of PD-L1 Expression in Patients With Soft Tissue Sarcoma. BMC Cancer (2016) 16:434. doi: 10.1186/s12885-016-2451-6 27393385 PMC4938996

[110] Zheng C You W Wan P Jiang X Chen J Zheng Y. Clinicopathological and Prognostic Significance of PD-L1 Expression in Sarcoma: A Systematic Review and Meta-Analysis. Med (Baltimore) (2018) 97:e11004. doi: 10.1097/MD.0000000000011004 PMC6024480 29923984

[111] Wang F Yu T Ma C Yuan H Zhang H Zhang Z. Prognostic Value of Programmed Cell Death 1 Ligand-1 in Patients With Bone and Soft Tissue Sarcomas: A Systemic and Comprehensive Meta-Analysis Based on 3,680 Patients. Front Oncol (2020) 10:749. doi: 10.3389/fonc.2020.00749 32582532 PMC7280448

[112] Zhu Z Jin Z Zhang M Tang Y Yang G Yuan X. Prognostic Value of Programmed Death-Ligand 1 in Sarcoma: A Meta-Analysis. Oncotarget (2017) 8:59570–80. doi: 10.18632/oncotarget.19168 PMC5601756 28938660

[113] Keung EZ Burgess M Salazar R Parra ER Rodrigues-Canales J Bolejack V. Correlative Analyses of the SARC028 Trial Reveal an Association Between Sarcoma-Associated Immune Infiltrate and Response to Pembrolizumab. Clin Cancer Res (2020) 26:1258–66. doi: 10.1158/1078-0432.CCR-19-1824 PMC7731262 31900276

[114] Italiano A Bessede A Bompas E Piperno-Neumann S Chevreau C Penel N. PD1 Inhibition in Soft-Tissue Sarcomas With Tertiary Lymphoid Structures: A Multicenter Phase II Trial. J Clin Oncol (2021) 39:11507–7. doi: 10.1200/JCO.2021.39.15_suppl.11507

[115] Vanhersecke L Brunet M Guégan J-P Rey C Bougouin A Cousin S. Mature Tertiary Lymphoid Structures Predict Immune Checkpoint Inhibitor Efficacy in Solid Tumors Independently of PD-L1 Expression. Nat Cancer (2021) 2:794–802. doi: 10.1038/s43018-021-00232-6 PMC8809887 35118423

[116] Vlenterie M Litière S Rizzo E Marréaud S Judson I Gelderblom H. Outcome of Chemotherapy in Advanced Synovial Sarcoma Patients: Review of 15 Clinical Trials From the European Organisation for Research and Treatment of Cancer Soft Tissue and Bone Sarcoma Group; Setting a New Landmark for Studies in This Entity. Eur J Cancer Oxf Engl 1990 (2016) 58:62–72. doi: 10.1016/j.ejca.2016.02.002 26968015

[117] Schöffski P Wozniak A Kasper B Aamdal S Leahy MG Rutkowski P. Activity and Safety of Crizotinib in Patients With Alveolar Soft Part Sarcoma With Rearrangement of TFE3: European Organization for Research and Treatment of Cancer (EORTC) Phase II Trial 90101 “CREATE.” Ann Oncol (2018) 29:758–65. doi: 10.1093/annonc/mdx774 29216400

[118] Martin-Broto J Stacchiotti S Lopez-Pousa A Redondo A Bernabeu D de Alava E. Pazopanib for Treatment of Advanced Malignant and Dedifferentiated Solitary Fibrous Tumour: A Multicentre, Single-Arm, Phase 2 Trial. Lancet Oncol (2019) 20:134–44. doi: 10.1016/S1470-2045(18)30676-4 30578023

[119] Stacchiotti S Ferrari S Redondo A Hindi N Palmerini E Vaz Salgado MA. Pazopanib for Treatment of Advanced Extraskeletal Myxoid Chondrosarcoma: A Multicentre, Single-Arm, Phase 2 Trial. Lancet Oncol (2019) 20:1252–62. doi: 10.1016/S1470-2045(19)30319-5 31331701

[120] Sleijfer S Ray-Coquard I Papai Z Le Cesne A Scurr M Schöffski P. Pazopanib, a Multikinase Angiogenesis Inhibitor, in Patients With Relapsed or Refractory Advanced Soft Tissue Sarcoma: A Phase II Study From the European Organisation for Research and Treatment of Cancer-Soft Tissue and Bone Sarcoma Group (EORTC Study 62043). J Clin Oncol (2009) 27:3126–32. doi: 10.1200/JCO.2008.21.3223 19451427

[121] Yang J Yan J Liu B. Targeting VEGF/VEGFR to Modulate Antitumor Immunity. Front Immunol (2018) 9:978. doi: 10.3389/fimmu.2018.00978 29774034 PMC5943566

[122] Fukumura D Kloepper J Amoozgar Z Duda DG Jain RK. Enhancing Cancer Immunotherapy Using Antiangiogenics: Opportunities and Challenges. Nat Rev Clin Oncol (2018) 15:325–40. doi: 10.1038/nrclinonc.2018.29 PMC5921900 29508855

[123] Atkins MB Plimack ER Puzanov I Fishman MN McDermott DF Cho DC. Axitinib in Combination With Pembrolizumab in Patients With Advanced Renal Cell Cancer: A non-Randomised, Open-Label, Dose-Finding, and Dose-Expansion Phase 1b Trial. Lancet Oncol (2018) 19:405–15. doi: 10.1016/S1470-2045(18)30081-0 PMC6860026 29439857

[124] Wilky BA Trucco MM Subhawong TK Florou V Park W Kwon D. Axitinib Plus Pembrolizumab in Patients With Advanced Sarcomas Including Alveolar Soft-Part Sarcoma: A Single-Centre, Single-Arm, Phase 2 Trial. Lancet Oncol (2019) 20:837–48. doi: 10.1016/S1470-2045(19)30153-6 31078463

[125] Martin-Broto J Hindi N Grignani G Martinez-Trufero J Redondo A Valverde C. Nivolumab and Sunitinib Combination in Advanced Soft Tissue Sarcomas: A Multicenter, Single-Arm, Phase Ib/II Trial. J Immunother Cancer (2020) 8. doi: 10.1136/jitc-2020-001561 PMC7674086 33203665

[126] Fumet J-D Limagne E Thibaudin M Ghiringhelli F. Immunogenic Cell Death and Elimination of Immunosuppressive Cells: A Double-Edged Sword of Chemotherapy. Cancers (2020) 12. doi: 10.3390/cancers12092637 PMC7565832 32947882

[127] Wu J Waxman DJ. Immunogenic Chemotherapy: Dose and Schedule Dependence and Combination With Immunotherapy. Cancer Lett (2018) 419:210–21. doi: 10.1016/j.canlet.2018.01.050 PMC5818299 29414305

[128] Pollack SM Ingham M Spraker MB Schwartz GK. Emerging Targeted and Immune-Based Therapies in Sarcoma. J Clin Oncol (2018) 36:125–35. doi: 10.1200/JCO.2017.75.1610 29220291

[129] Galluzzi L Humeau J Buqué A Zitvogel L Kroemer G. Immunostimulation With Chemotherapy in the Era of Immune Checkpoint Inhibitors. Nat Rev Clin Oncol (2020) 17:725–41. doi: 10.1038/s41571-020-0413-z 32760014

[130] Chan AS Ng VY Snider J Kallen ME Miller KD. Hyperprogression of Liver Metastasis With Neoadjuvant Immunotherapy for Soft Tissue Sarcoma. Cureus (2020) 12:e8575. doi: 10.7759/cureus.8575 32670711 PMC7358953

[131] Rd I Lh L JV R W P R P W P G. Effect of Neoadjuvant Chemotherapy Plus Regional Hyperthermia on Long-Term Outcomes Among Patients With Localized High-Risk Soft Tissue Sarcoma: The EORTC 62961-ESHO 95 Randomized Clinical Trial. JAMA Oncol (2018) 4. doi: 10.1001/jamaoncol.2017.4996 PMC5885262 29450452

[132] Issels R Büclein V Kampmann E Knösel T Nössner E Subklewe M. Dissecting the Role of Tumor-Infiltrating Lymphocytes (TIL) in Patients With High-Risk Soft-Tissue Sarcoma (STS) Receiving Neo-Adjuvant Chemotherapy (NAC) With Regional Hyperthermia (RHT). Ann Oncol (2016) 27:vi488. doi: 10.1093/annonc/mdw388.18

[133] Ryan CW Merimsky O Agulnik M Blay J-Y Schuetze SM Van Tine BA. PICASSO III: A Phase III, Placebo-Controlled Study of Doxorubicin With or Without Palifosfamide in Patients With Metastatic Soft Tissue Sarcoma. J Clin Oncol (2016) 34:3898–905. doi: 10.1200/JCO.2016.67.6684 27621408

[134] Tap WD Papai Z Van Tine BA Attia S Ganjoo KN Jones RL. Doxorubicin Plus Evofosfamide Versus Doxorubicin Alone in Locally Advanced, Unresectable or Metastatic Soft-Tissue Sarcoma (TH CR-406/SARC021): An International, Multicentre, Open-Label, Randomised Phase 3 Trial. Lancet Oncol (2017) 18:1089–103. doi: 10.1016/S1470-2045(17)30381-9 PMC7771354 28651927

[135] Mattarollo SR Loi S Duret H Ma Y Zitvogel L Smyth MJ. Pivotal Role of Innate and Adaptive Immunity in Anthracycline Chemotherapy of Established Tumors. Cancer Res (2011) 71:4809–20. doi: 10.1158/0008-5472.CAN-11-0753 21646474

[136] Pollack SM Redman MW Baker KK Wagner MJ Schroeder BA Loggers ET. Assessment of Doxorubicin and Pembrolizumab in Patients With Advanced Anthracycline-Naive Sarcoma: A Phase 1/2 Nonrandomized Clinical Trial. JAMA Oncol (2020) 6:1778–82. doi: 10.1001/jamaoncol.2020.3689 PMC7489365 32910151

[137] Livingston MB Jagosky M Robinson MM Ahrens W Benbow JH Farhangfar CJ. A Pilot Study Evaluating the Safety, Tolerability, and Efficacy of Doxorubicin and Pembrolizumab in Patients With Metastatic or Unresectable Soft Tissue Sarcoma. J Clin Oncol (2020) 38:11519–9. doi: 10.1200/JCO.2020.38.15_suppl.11519

[138] Germano G Frapolli R Belgiovine C Anselmo A Pesce S Liguori M. Role of Macrophage Targeting in the Antitumor Activity of Trabectedin. Cancer Cell (2013) 23:249–62. doi: 10.1016/j.ccr.2013.01.008 23410977

[139] Demetri GD von Mehren M Jones RL Hensley ML Schuetze SM Staddon A. Efficacy and Safety of Trabectedin or Dacarbazine for Metastatic Liposarcoma or Leiomyosarcoma After Failure of Conventional Chemotherapy: Results of a Phase III Randomized Multicenter Clinical Trial. J Clin Oncol (2016) 34:786–93. doi: 10.1200/JCO.2015.62.4734 PMC5070559 26371143

[140] Gordon EM Chua-Alcala VS Kim K Dy PS Paz MK Angel N. SAINT: Results of an Expanded Phase II Study Using Safe Amounts of Ipilimumab (I), Nivolumab (N), and Trabectedin (T) as First-Line Treatment of Advanced Soft Tissue Sarcoma [NCT03138161]. J Clin Oncol (2020) 38:11520–0. doi: 10.1200/JCO.2020.38.15_suppl.11520

[141] Kawai A Araki N Sugiura H Ueda T Yonemoto T Takahashi M. Trabectedin Monotherapy After Standard Chemotherapy Versus Best Supportive Care in Patients With Advanced, Translocation-Related Sarcoma: A Randomised, Open-Label, Phase 2 Study. Lancet Oncol (2015) 16:406–16. doi: 10.1016/S1470-2045(15)70098-7 25795406

[142] Toulmonde M Brahmi M Giraud A Bessede A Kind M Toulza E. LBA67 TRAMUNE, a Phase Ib Study Combining Trabectedin and Durvalumab, Results of the Expansion Cohort in Patients With Advanced Pretreated Soft Tissue Sarcomas. Ann Oncol (2020) 31:S1199. doi: 10.1016/j.annonc.2020.08.2308

[143] Schöffski P Chawla S Maki RG Italiano A Gelderblom H Choy E. Eribulin Versus Dacarbazine in Previously Treated Patients With Advanced Liposarcoma or Leiomyosarcoma: A Randomised, Open-Label, Multicentre, Phase 3 Trial. Lancet Lond Engl (2016) 387:1629–37. doi: 10.1016/S0140-6736(15)01283-0 26874885

[144] Ito K Hamamichi S Abe T Akagi T Shirota H Kawano S. Antitumor Effects of Eribulin Depend on Modulation of the Tumor Microenvironment by Vascular Remodeling in Mouse Models. Cancer Sci (2017) 108:2273–80. doi: 10.1111/cas.13392 PMC5665763 28869796

[145] Dybdal-Hargreaves NF Risinger AL Mooberry SL. Eribulin Mesylate: Mechanism of Action of a Unique Microtubule Targeting Agent. Clin Cancer Res (2015) 21:2445–52. doi: 10.1158/1078-0432.CCR-14-3252 PMC4812567 25838395

[146] Nathenson M Choy E Carr ND Hibbard HD Mazzola E Catalano PJ. Phase II Study of Eribulin and Pembrolizumab in Patients (Pts) With Metastatic Soft Tissue Sarcomas (STS): Report of LMS Cohort. J Clin Oncol (2020) 38:11559–9. doi: 10.1200/JCO.2020.38.15_suppl.11559

[147] Aznar MA Tinari N Rullán AJ Sánchez-Paulete AR Rodriguez-Ruiz ME Melero I. Intratumoral Delivery of Immunotherapy-Act Locally, Think Globally. J Immunol Baltim Md 1950 (2017) 198:31–9. doi: 10.4049/jimmunol.1601145 27994166

[148] Marabelle A Tselikas L de Baere T Houot R. Intratumoral Immunotherapy: Using the Tumor as the Remedy. Ann Oncol (2017) 28:xii33–43. doi: 10.1093/annonc/mdx683 29253115

[149] Marabelle A Andtbacka R Harrington K Melero I Leidner R de Baere T. Starting the Fight in the Tumor: Expert Recommendations for the Development of Human Intratumoral Immunotherapy (HIT-It). Ann Oncol (2018) 29:2163–74. doi: 10.1093/annonc/mdy423 PMC6290929 30295695

[150] Raj S Miller LD Triozzi PL. Addressing the Adult Soft Tissue Sarcoma Microenvironment With Intratumoral Immunotherapy. Sarcoma (2018) 2018:9305294. doi: 10.1155/2018/9305294 30158830 PMC6109466

[151] Lichty BD Breitbach CJ Stojdl DF Bell JC. Going Viral With Cancer Immunotherapy. Nat Rev Cancer (2014) 14:559–67. doi: 10.1038/nrc3770 24990523

[152] Everts A Bergeman M McFadden G Kemp V. Simultaneous Tumor and Stroma Targeting by Oncolytic Viruses. Biomedicines (2020) 8. doi: 10.3390/biomedicines8110474 PMC7694393 33167307

[153] Tazawa H Hasei J Yano S Kagawa S Ozaki T Fujiwara T. Bone and Soft-Tissue Sarcoma: A New Target for Telomerase-Specific Oncolytic Virotherapy. Cancers (2020) 12. doi: 10.3390/cancers12020478 PMC7072448 32085583

[154] Zhang B Cheng P. Improving Antitumor Efficacy *via* Combinatorial Regimens of Oncolytic Virotherapy. Mol Cancer (2020) 19:158. doi: 10.1186/s12943-020-01275-6 33172438 PMC7656670

[155] Smith HG Mansfield D Roulstone V Kyula-Currie JN McLaughlin M Patel RR. PD-1 Blockade Following Isolated Limb Perfusion With Vaccinia Virus Prevents Local and Distant Relapse of Soft-Tissue Sarcoma. Clin Cancer Res (2019) 25:3443–54. doi: 10.1158/1078-0432.CCR-18-3767 30885937

[156] Cinatl J Cinatl J Michaelis M Kabickova H Kotchetkov R Vogel J-U. Potent Oncolytic Activity of Multimutated Herpes Simplex Virus G207 in Combination With Vincristine Against Human Rhabdomyosarcoma. Cancer Res (2003) 63:1508–14. 12670897

[157] Siurala M Bramante S Vassilev L Hirvinen M Parviainen S Tähtinen S. Oncolytic Adenovirus and Doxorubicin-Based Chemotherapy Results in Synergistic Antitumor Activity Against Soft-Tissue Sarcoma. Int J Cancer (2015) 136:945–54. doi: 10.1002/ijc.29048 24975392

[158] Ribas A Dummer R Puzanov I VanderWalde A Andtbacka RHI Michielin O. Oncolytic Virotherapy Promotes Intratumoral T Cell Infiltration and Improves Anti-PD-1 Immunotherapy. Cell (2017) 170:1109–19.e10. doi: 10.1016/j.cell.2017.08.027 28886381 PMC8034392

[159] Galanis E Okuno SH Nascimento AG Lewis BD Lee RA Oliveira AM. Phase I-II Trial of ONYX-015 in Combination With MAP Chemotherapy in Patients With Advanced Sarcomas. Gene Ther (2005) 12:437–45. doi: 10.1038/sj.gt.3302436 15647767

[160] Kelly CM Antonescu CR Bowler T Munhoz R Chi P Dickson MA. Objective Response Rate Among Patients With Locally Advanced or Metastatic Sarcoma Treated With Talimogene Laherparepvec in Combination With Pembrolizumab: A Phase 2 Clinical Trial. JAMA Oncol (2020) 6:402–8. doi: 10.1001/jamaoncol.2019.6152 PMC6990941 31971541

[161] Andtbacka RHI Kaufman HL Collichio F Amatruda T Senzer N Chesney J. Talimogene Laherparepvec Improves Durable Response Rate in Patients With Advanced Melanoma. J Clin Oncol (2015) 33:2780–8. doi: 10.1200/JCO.2014.58.3377 26014293

[162] Apostolopoulos V. Cancer Vaccines: Research and Applications. Cancers (2019) 11. doi: 10.3390/cancers11081041 PMC6721783 31344788

[163] Igarashi Y Sasada T. Cancer Vaccines: Toward the Next Breakthrough in Cancer Immunotherapy. J Immunol Res (2020) 2020:5825401. doi: 10.1155/2020/5825401 33282961 PMC7685825

[164] Dhodapkar MV Sznol M Zhao B Wang D Carvajal RD Keohan ML. Induction of Antigen-Specific Immunity With a Vaccine Targeting NY-ESO-1 to the Dendritic Cell Receptor DEC-205. Sci Transl Med (2014) 6:232ra51. doi: 10.1126/scitranslmed.3008068 PMC6151129 24739759

[165] Somaiah N Block MS Kim JW Shapiro GI Do KT Hwu P. First-In-Class, First-In-Human Study Evaluating LV305, a Dendritic-Cell Tropic Lentiviral Vector, in Sarcoma and Other Solid Tumors Expressing NY-ESO-1. Clin Cancer Res (2019) 25:5808–17. doi: 10.1158/1078-0432.CCR-19-1025 31227504

[166] Chawla S Tine BAV Pollack S Ganjoo K Elias A Riedel RF. A Phase 2 Study of CMB305 and Atezolizumab in NY-ESO-1+ Soft Tissue Sarcoma: Interim Analysis of Immunogenicity, Tumor Control and Survival. Ann Oncol (2017) 28:v523. doi: 10.1093/annonc/mdx387.007

[167] Jäger E Gnjatic S Nagata Y Stockert E Jäger D Karbach J. Induction of Primary NY-ESO-1 Immunity: CD8+ T Lymphocyte and Antibody Responses in Peptide-Vaccinated Patients With NY-ESO-1+ Cancers. Proc Natl Acad Sci U S A (2000) 97:12198–203. doi: 10.1073/pnas.220413497 PMC17318 11027314

[168] Davis ID Chen W Jackson H Parente P Shackleton M Hopkins W. Recombinant NY-ESO-1 Protein With ISCOMATRIX Adjuvant Induces Broad Integrated Antibody and CD4(+) and CD8(+) T Cell Responses in Humans. Proc Natl Acad Sci U S A (2004) 101:10697–702. doi: 10.1073/pnas.0403572101 PMC489997 15252201

[169] Sato Y Nabeta Y Tsukahara T Hirohashi Y Syunsui R Maeda A. Detection and Induction of CTLs Specific for SYT-SSX-Derived Peptides in HLA-A24(+) Patients With Synovial Sarcoma. J Immunol Baltim Md 1950 (2002) 169:1611–8. doi: 10.4049/jimmunol.169.3.1611 12133991

[170] Kawaguchi S Tsukahara T Ida K Kimura S Murase M Kano M. SYT-SSX Breakpoint Peptide Vaccines in Patients With Synovial Sarcoma: A Study From the Japanese Musculoskeletal Oncology Group. Cancer Sci (2012) 103:1625–30. doi: 10.1111/j.1349-7006.2012.02370.x PMC7659233 22726592

[171] Robbins PF Kassim SH Tran TLN Crystal JS Morgan RA Feldman SA. A Pilot Trial Using Lymphocytes Genetically Engineered With an NY-ESO-1-Reactive T-Cell Receptor: Long-Term Follow-Up and Correlates With Response. Clin Cancer Res (2015) 21:1019–27. doi: 10.1158/1078-0432.CCR-14-2708 PMC4361810 25538264

[172] D’Angelo SP Melchiori L Merchant MS Bernstein D Glod J Kaplan R. Antitumor Activity Associated With Prolonged Persistence of Adoptively Transferred NY-ESO-1 C259t Cells in Synovial Sarcoma. Cancer Discov (2018) 8:944–57. doi: 10.1158/2159-8290.CD-17-1417 PMC8092079 29891538

[173] Ramachandran I Lowther DE Dryer-Minnerly R Wang R Fayngerts S Nunez D. Systemic and Local Immunity Following Adoptive Transfer of NY-ESO-1 SPEAR T Cells in Synovial Sarcoma. J Immunother Cancer (2019) 7:276. doi: 10.1186/s40425-019-0762-2 31651363 PMC6813983

[174] D’Angelo S Demetri G Tine BV Druta M Glod J Chow W. 298 Final Analysis of the Phase 1 Trial of NY-ESO-1–Specific T-Cell Receptor (TCR) T-Cell Therapy (Letetresgene Autoleucel; GSK3377794) in Patients With Advanced Synovial Sarcoma (SS). J Immunother Cancer (2020) 8. doi: 10.1136/jitc-2020-SITC2020.0298

[175] Tine BAV Butler MO Araujo D Johnson ML Clarke J Liebner D. ADP-A2M4 (MAGE-A4) in Patients With Synovial Sarcoma. Ann Oncol (2019) 30:v684–5. doi: 10.1093/annonc/mdz283.003

[176] Thanindratarn P Dean DC Nelson SD Hornicek FJ Duan Z. Chimeric Antigen Receptor T (CAR-T) Cell Immunotherapy for Sarcomas: From Mechanisms to Potential Clinical Applications. Cancer Treat Rev (2020) 82:101934. doi: 10.1016/j.ctrv.2019.101934 31794912

[177] Ahmed N Brawley VS Hegde M Robertson C Ghazi A Gerken C. Human Epidermal Growth Factor Receptor 2 (HER2) -Specific Chimeric Antigen Receptor-Modified T Cells for the Immunotherapy of HER2-Positive Sarcoma. J Clin Oncol (2015) 33:1688–96. doi: 10.1200/JCO.2014.58.0225 PMC4429176 25800760

[178] Ducimetière F Lurkin A Ranchère-Vince D Decouvelaere A-V Péoc’h M Istier L. Incidence of Sarcoma Histotypes and Molecular Subtypes in a Prospective Epidemiological Study With Central Pathology Review and Molecular Testing. PLoS One (2011) 6:e20294. doi: 10.1371/journal.pone.0020294 21826194 PMC3149593

[179] Lee PJ Yoo NS Hagemann IS Pfeifer JD Cottrell CE Abel HJ. Spectrum of Mutations in Leiomyosarcomas Identified by Clinical Targeted Next-Generation Sequencing. Exp Mol Pathol (2017) 102:156–61. doi: 10.1016/j.yexmp.2017.01.012 28093192

[180] Hernando E Charytonowicz E Dudas ME Menendez S Matushansky I Mills J. The AKT-mTOR Pathway Plays a Critical Role in the Development of Leiomyosarcomas. Nat Med (2007) 13:748–53. doi: 10.1038/nm1560 17496901

[181] Oza J Doshi SD Hao L Musi E Schwartz GK Ingham M. Homologous Recombination Repair Deficiency as a Therapeutic Target in Sarcoma. Semin Oncol (2020) 47:380–9. doi: 10.1053/j.seminoncol.2020.10.002 33183763

[182] Leitao MM Hensley ML Barakat RR Aghajanian C Gardner GJ Jewell EL. Immunohistochemical Expression of Estrogen and Progesterone Receptors and Outcomes in Patients With Newly Diagnosed Uterine Leiomyosarcoma. Gynecol Oncol (2012) 124:558–62. doi: 10.1016/j.ygyno.2011.11.009 22085894

[183] Seddon B Scurr M Jones RL Wood Z Propert-Lewis C Fisher C. A Phase II Trial to Assess the Activity of Gemcitabine and Docetaxel as First Line Chemotherapy Treatment in Patients With Unresectable Leiomyosarcoma. Clin Sarcoma Res (2015) 5:13. doi: 10.1186/s13569-015-0029-8 25987978 PMC4434867

[184] van der Graaf WTA Blay J-Y Chawla SP Kim D-W Bui-Nguyen B Casali PG. Pazopanib for Metastatic Soft-Tissue Sarcoma (PALETTE): A Randomised, Double-Blind, Placebo-Controlled Phase 3 Trial. Lancet Lond Engl (2012) 379:1879–86. doi: 10.1016/S0140-6736(12)60651-5 22595799

[185] Martin-Broto J Pousa AL de Las Peñas R García Del Muro X Gutierrez A Martinez-Trufero J. Randomized Phase II Study of Trabectedin and Doxorubicin Compared With Doxorubicin Alone as First-Line Treatment in Patients With Advanced Soft Tissue Sarcomas: A Spanish Group for Research on Sarcoma Study. J Clin Oncol (2016) 34:2294–302. doi: 10.1200/JCO.2015.65.3329 27185843

[186] Wunder JS Lee MJ Nam J Lau BY Dickson BC Pinnaduwage D. Osteosarcoma and Soft-Tissue Sarcomas With an Immune Infiltrate Express PD-L1: Relation to Clinical Outcome and Th1 Pathway Activation. Oncoimmunology (2020) 9:1737385. doi: 10.1080/2162402X.2020.1737385 33457085 PMC7790526

[187] Lee C-H Espinosa I Vrijaldenhoven S Subramanian S Montgomery KD Zhu S. Prognostic Significance of Macrophage Infiltration in Leiomyosarcomas. Clin Cancer Res (2008) 14:1423–30. doi: 10.1158/1078-0432.CCR-07-1712 18316565

[188] George S Miao D Demetri GD Adeegbe D Rodig SJ Shukla S. Loss of PTEN Is Associated With Resistance to Anti-PD-1 Checkpoint Blockade Therapy in Metastatic Uterine Leiomyosarcoma. Immunity (2017) 46:197–204. doi: 10.1016/j.immuni.2017.02.001 28228279 PMC5408320

[189] Peng W Chen JQ Liu C Malu S Creasy C Tetzlaff MT. Loss of PTEN Promotes Resistance to T Cell-Mediated Immunotherapy. Cancer Discov (2016) 6:202–16. doi: 10.1158/2159-8290.CD-15-0283 PMC4744499 26645196

[190] Iwasaki T Kohashi K Toda Y Ishihara S Yamada Y Oda Y. Correction to: Association of PD-L1 and IDO1 Expression With JAK-STAT Pathway Activation in Soft-Tissue Leiomyosarcoma. J Cancer Res Clin Oncol (2020) 147:1451–63. doi: 10.1007/s00432-020-03466-6 PMC11801941 32951108

[191] Xi C Wencheng Z Dong Q Yong G Cihui Y Yuwen W. Tumor Regression After Combination of Radiation and PD-1 Antibody Nivolumab Treatment in a Patient With Metastatic Mediastinal Leiomyosarcoma: A Case Report. Cancer Biol Ther (2019) 20:408–12. doi: 10.1080/15384047.2018.1537577 PMC6422505 30388926

[192] Yang L Chen S Luo P Yan W Wang C. Liposarcoma: Advances in Cellular and Molecular Genetics Alterations and Corresponding Clinical Treatment. J Cancer (2020) 11:100–7. doi: 10.7150/jca.36380 PMC6930414 31892977

[193] Bill KLJ Casadei L Prudner BC Iwenofu H Strohecker AM Pollock RE. Liposarcoma: Molecular Targets and Therapeutic Implications. Cell Mol Life Sci CMLS (2016) 73:3711–8. doi: 10.1007/s00018-016-2266-2 PMC7175098 27173057

[194] Kanojia D Nagata Y Garg M Lee DH Sato A Yoshida K. Genomic Landscape of Liposarcoma. Oncotarget (2015) 6:42429–44. doi: 10.18632/oncotarget.6464 PMC4767443 26643872

[195] Jones RL Fisher C Al-Muderis O Judson IR. Differential Sensitivity of Liposarcoma Subtypes to Chemotherapy. Eur J Cancer Oxf Engl 1990 (2005) 41:2853–60. doi: 10.1016/j.ejca.2005.07.023 16289617

[196] Italiano A Toulmonde M Cioffi A Penel N Isambert N Bompas E. Advanced Well-Differentiated/Dedifferentiated Liposarcomas: Role of Chemotherapy and Survival. Ann Oncol (2012) 23:1601–7. doi: 10.1093/annonc/mdr485 22039081

[197] Livingston JA Bugano D Barbo A Lin H Madewell JE Wang WL. Role of Chemotherapy in Dedifferentiated Liposarcoma of the Retroperitoneum: Defining the Benefit and Challenges of the Standard. Sci Rep (2017) 7:11836. doi: 10.1038/s41598-017-12132-w 28928422 PMC5605500

[198] Lansu J Bovée JVMG Braam P van Boven H Flucke U Bonenkamp JJ. Dose Reduction of Preoperative Radiotherapy in Myxoid Liposarcoma: A Nonrandomized Controlled Trial. JAMA Oncol (2021) 7:e205865. doi: 10.1001/jamaoncol.2020.5865 33180100 PMC7662477

[199] Grosso F Jones RL Demetri GD Judson IR Blay J-Y Le Cesne A. Efficacy of Trabectedin (Ecteinascidin-743) in Advanced Pretreated Myxoid Liposarcomas: A Retrospective Study. Lancet Oncol (2007) 8:595–602. doi: 10.1016/S1470-2045(07)70175-4 17586092

[200] Jeon HM Lee JS Kim SH Yun K-H Park KH Jeon MK. Comprehensive Immuno-Molecular Profiles for Liposarcoma: Roles of Programmed Death Ligand 1, Microsatellite Instability, and PIK3CA. Oncology (2020) 98:817–26. doi: 10.1159/000509004 32892196

[201] Yan L Wang Z Cui C Guan X Dong B Zhao M. Comprehensive Immune Characterization and T-Cell Receptor Repertoire Heterogeneity of Retroperitoneal Liposarcoma. Cancer Sci (2019) 110:3038–48. doi: 10.1111/cas.14161 PMC6778648 31385405

[202] Miyake M Oda Y Nishimura N Morizawa Y Ohnishi S Hatakeyama K. Integrative Assessment of Clinicopathological Parameters and the Expression of PD-L1, PD-L2 and PD-1 in Tumor Cells of Retroperitoneal Sarcoma. Oncol Lett (2020) 20:190. doi: 10.3892/ol.2020.12052 32952659 PMC7479533

[203] Tseng WW Malu S Zhang M Chen J Sim GC Wei W. Analysis of the Intratumoral Adaptive Immune Response in Well Differentiated and Dedifferentiated Retroperitoneal Liposarcoma. Sarcoma (2015) 2015:547460. doi: 10.1155/2015/547460 25705114 PMC4326351

[204] Nabeshima A Matsumoto Y Fukushi J Iura K Matsunobu T Endo M. Tumour-Associated Macrophages Correlate With Poor Prognosis in Myxoid Liposarcoma and Promote Cell Motility and Invasion *via* the HB-EGF-EGFR-PI3K/Akt Pathways. Br J Cancer (2015) 112:547–55. doi: 10.1038/bjc.2014.637 PMC4453656 25562433

[205] Ray-Coquard I Blay J-Y Italiano A Le Cesne A Penel N Zhi J. Effect of the MDM2 Antagonist RG7112 on the P53 Pathway in Patients With MDM2-Amplified, Well-Differentiated or Dedifferentiated Liposarcoma: An Exploratory Proof-of-Mechanism Study. Lancet Oncol (2012) 13:1133–40. doi: 10.1016/S1470-2045(12)70474-6 23084521

[206] Zang H Peng J Zheng H Fan S. Hyperprogression After Immune-Checkpoint Inhibitor Treatment: Characteristics and Hypotheses. Front Oncol (2020) 10:515. doi: 10.3389/fonc.2020.00515 32411591 PMC7201048

[207] Sahin I Zhang S Navaraj A Zhou L Dizon D Safran H. AMG-232 Sensitizes High MDM2-Expressing Tumor Cells to T-Cell-Mediated Killing. Cell Death Discov (2020) 6:57. doi: 10.1038/s41420-020-0292-1 32655895 PMC7338458

[208] Veneziani I Infante P Ferretti E Melaiu O Battistelli C Lucarini V. Nutlin-3a Enhances Natural Killer Cell-Mediated Killing of Neuroblastoma by Restoring P53-Dependent Expression of Ligands for NKG2D and DNAM-1 Receptors. Cancer Immunol Res (2020) 9:170–83. doi: 10.1158/2326-6066.CIR-20-0313 33303573

[209] Fang DD Tang Q Kong Y Wang Q Gu J Fang X. MDM2 Inhibitor APG-115 Synergizes With PD-1 Blockade Through Enhancing Antitumor Immunity in the Tumor Microenvironment. J Immunother Cancer (2019) 7:327. doi: 10.1186/s40425-019-0750-6 31779710 PMC6883539

[210] Dickson MA Schwartz GK Keohan ML D’Angelo SP Gounder MM Chi P. Progression-Free Survival Among Patients With Well-Differentiated or Dedifferentiated Liposarcoma Treated With CDK4 Inhibitor Palbociclib: A Phase 2 Clinical Trial. JAMA Oncol (2016) 2:937–40. doi: 10.1001/jamaoncol.2016.0264 PMC4991028 27124835

[211] Goel S DeCristo MJ Watt AC BrinJones H Sceneay J Li BB. CDK4/6 Inhibition Triggers Anti-Tumour Immunity. Nature (2017) 548:471–5. doi: 10.1038/nature23465 PMC5570667 28813415

[212] Taylor BS DeCarolis PL Angeles CV Brenet F Schultz N Antonescu CR. Frequent Alterations and Epigenetic Silencing of Differentiation Pathway Genes in Structurally Rearranged Liposarcomas. Cancer Discov (2011) 1:587–97. doi: 10.1158/2159-8290.CD-11-0181 PMC3274776 22328974

[213] Aspeslagh S Morel D Soria J-C Postel-Vinay S. Epigenetic Modifiers as New Immunomodulatory Therapies in Solid Tumours. Ann Oncol (2018) 29:812–24. doi: 10.1093/annonc/mdy050 29432557

[214] D’Incalci M Galmarini CM. A Review of Trabectedin (ET-743): A Unique Mechanism of Action. Mol Cancer Ther (2010) 9:2157–63. doi: 10.1158/1535-7163.MCT-10-0263 20647340

[215] Wisdom AJ Mowery YM Riedel RF Kirsch DG. Rationale and Emerging Strategies for Immune Checkpoint Blockade in Soft Tissue Sarcoma. Cancer (2018) 124:3819–29. doi: 10.1002/cncr.31517 PMC6215523 29723407

[216] Gronchi A Hindi N Cruz J Blay J-Y Lopez-Pousa A Italiano A. Trabectedin and RAdiotherapy in Soft Tissue Sarcoma (TRASTS): Results of a Phase I Study in Myxoid Liposarcoma From Spanish (GEIS), Italian (ISG), French (FSG) Sarcoma Groups. EClinicalMedicine (2019) 9:35–43. doi: 10.1016/j.eclinm.2019.03.007 31143880 PMC6510725

[217] Weiss SW Enzinger FM. Malignant Fibrous Histiocytoma. An Analysis of 200 Cases. Cancer (1978) 41:2250–66. doi: 10.1002/1097-0142(197806)41:6<2250::AID-CNCR2820410626>3.0.CO;2-W 207408

[218] Delisca GO Mesko NW Alamanda VK Archer KR Song Y Halpern JL. MFH and High-Grade Undifferentiated Pleomorphic Sarcoma-What’s in a Name? J Surg Oncol (2015) 111:173–7. doi: 10.1002/jso.23787 25219789

[219] Kamat NV Million L Yao D-H Donaldson SS Mohler DG van de Rijn M. The Outcome of Patients With Localized Undifferentiated Pleomorphic Sarcoma of the Lower Extremity Treated at Stanford University. Am J Clin Oncol (2019) 42:166–71. doi: 10.1097/COC.0000000000000496 30557163

[220] Kelleher FC Viterbo A. Histologic and Genetic Advances in Refining the Diagnosis of “Undifferentiated Pleomorphic Sarcoma.” Cancers (2013) 5:218–33. doi: 10.3390/cancers5010218 PMC3730306 24216705

[221] Lewin J Garg S Lau BY Dickson BC Traub F Gokgoz N. Identifying Actionable Variants Using Next Generation Sequencing in Patients With a Historical Diagnosis of Undifferentiated Pleomorphic Sarcoma. Int J Cancer (2018) 142:57–65. doi: 10.1002/ijc.31039 28891048

[222] Fletcher CD. Pleomorphic Malignant Fibrous Histiocytoma: Fact or Fiction? A Critical Reappraisal Based on 159 Tumors Diagnosed as Pleomorphic Sarcoma. Am J Surg Pathol (1992) 16:213–28. 1317996

[223] Konstantinopoulos PA Fountzilas E Goldsmith JD Bhasin M Pillay K Francoeur N. Analysis of Multiple Sarcoma Expression Datasets: Implications for Classification, Oncogenic Pathway Activation and Chemotherapy Resistance. PLoS One (2010) 5:e9747. doi: 10.1371/journal.pone.0009747 20368975 PMC2848563

[224] Canter RJ Beal S Borys D Martinez SR Bold RJ Robbins AS. Interaction of Histologic Subtype and Histologic Grade in Predicting Survival for Soft-Tissue Sarcomas. J Am Coll Surg (2010) 210:191–8.e2. doi: 10.1016/j.jamcollsurg.2009.10.007 20113939

[225] Zheng B Qu Y Wang J Shi Y Yan W. Pathogenic and Targetable Genetic Alterations in Resected Recurrent Undifferentiated Pleomorphic Sarcomas Identified by Targeted Next-Generation Sequencing. Cancer Genomics Proteomics (2019) 16:221–8. doi: 10.21873/cgp.20127 PMC6542646 31018952

[226] Rüping K Altendorf-Hofmann A Chen Y Kampmann E Gibis S Lindner L. High IGF2 and FGFR3 are Associated With Tumour Progression in Undifferentiated Pleomorphic Sarcomas, But EGFR and FGFR3 Mutations are a Rare Event. J Cancer Res Clin Oncol (2014) 140:1315–22. doi: 10.1007/s00432-014-1700-9 PMC11823920 24804818

[227] Toulmonde M Lucchesi C Verbeke S Crombe A Adam J Geneste D. High Throughput Profiling of Undifferentiated Pleomorphic Sarcomas Identifies Two Main Subgroups With Distinct Immune Profile, Clinical Outcome and Sensitivity to Targeted Therapies. EBioMedicine (2020) 62:103131. doi: 10.1016/j.ebiom.2020.103131 33254023 PMC7708794

[228] Wong P Hui A Su J Yue S Haibe-Kains B Gokgoz N. Prognostic microRNAs Modulate the RHO Adhesion Pathway: A Potential Therapeutic Target in Undifferentiated Pleomorphic Sarcomas. Oncotarget (2015) 6:39127–39. doi: 10.18632/oncotarget.3926 PMC4770761 25970788

[229] Baird K Davis S Antonescu CR Harper UL Walker RL Chen Y. Gene Expression Profiling of Human Sarcomas: Insights Into Sarcoma Biology. Cancer Res (2005) 65:9226–35. doi: 10.1158/0008-5472.CAN-05-1699 16230383

[230] Serrano C Romagosa C Hernández-Losa J Simonetti S Valverde C Moliné T. RAS/MAPK Pathway Hyperactivation Determines Poor Prognosis in Undifferentiated Pleomorphic Sarcomas. Cancer (2016) 122:99–107. doi: 10.1002/cncr.29733 26479291

[231] Roland CL May CD Watson KL Al Sannaa GA Dineen SP Feig R. Analysis of Clinical and Molecular Factors Impacting Oncologic Outcomes in Undifferentiated Pleomorphic Sarcoma. Ann Surg Oncol (2016) 23:2220–8. doi: 10.1245/s10434-016-5115-5 PMC4937623 26847678

[232] Carneiro A Francis P Bendahl P-O Fernebro J Akerman M Fletcher C. Indistinguishable Genomic Profiles and Shared Prognostic Markers in Undifferentiated Pleomorphic Sarcoma and Leiomyosarcoma: Different Sides of a Single Coin? Lab Investig J Tech Methods Pathol (2009) 89:668–75. doi: 10.1038/labinvest.2009.18 19290004

[233] Villacis RAR Silveira SM Barros-Filho MC Marchi FA Domingues MAC Scapulatempo-Neto C. Gene Expression Profiling in Leiomyosarcomas and Undifferentiated Pleomorphic Sarcomas: SRC as a New Diagnostic Marker. PLoS One (2014) 9:e102281. doi: 10.1371/journal.pone.0102281 25028927 PMC4100821

[234] Silveira SM Villacis RAR Marchi FA Barros Filho M de C Drigo SA Neto CS. Genomic Signatures Predict Poor Outcome in Undifferentiated Pleomorphic Sarcomas and Leiomyosarcomas. PLoS One (2013) 8:e67643. doi: 10.1371/journal.pone.0067643 23825676 PMC3692486

[235] Gibault L Pérot G Chibon F Bonnin S Lagarde P Terrier P. New Insights in Sarcoma Oncogenesis: A Comprehensive Analysis of a Large Series of 160 Soft Tissue Sarcomas With Complex Genomics. J Pathol (2011) 223:64–71. doi: 10.1002/path.2787 21125665

[236] Lahat G Tuvin D Wei C Wang WL Pollock RE Anaya DA. Molecular Prognosticators of Complex Karyotype Soft Tissue Sarcoma Outcome: A Tissue Microarray-Based Study. Ann Oncol (2010) 21:1112–20. doi: 10.1093/annonc/mdp459 19875755

[237] Lahat G Zhang P Zhu Q-S Torres K Ghadimi M Smith KD. The Expression of C-Met Pathway Components in Unclassified Pleomorphic Sarcoma/Malignant Fibrous Histiocytoma (UPS/MFH): A Tissue Microarray Study. Histopathology (2011) 59:556–61. doi: 10.1111/j.1365-2559.2011.03946.x 22034893

[238] Takahashi A Nakayama R Ishibashi N Doi A Ichinohe R Ikuyo Y. Analysis of Gene Expression Profiles of Soft Tissue Sarcoma Using a Combination of Knowledge-Based Filtering With Integration of Multiple Statistics. PLoS One (2014) 9:e106801. doi: 10.1371/journal.pone.0106801 25188299 PMC4154757

[239] Chen L Oke T Siegel N Cojocaru G Tam AJ Blosser RL. The Immunosuppressive Niche of Soft-Tissue Sarcomas is Sustained by Tumor-Associated Macrophages and Characterized by Intratumoral Tertiary Lymphoid Structures. Clin Cancer Res (2020) 26:4018–30. doi: 10.1158/1078-0432.CCR-19-3416 PMC8772618 32332015

[240] Keung EZ Tsai J-W Ali AM Cormier JN Bishop AJ Guadagnolo BA. Analysis of the Immune Infiltrate in Undifferentiated Pleomorphic Sarcoma of the Extremity and Trunk in Response to Radiotherapy: Rationale for Combination Neoadjuvant Immune Checkpoint Inhibition and Radiotherapy. Oncoimmunology (2018) 7:e1385689. doi: 10.1080/2162402X.2017.1385689 29308306 PMC5749668

[241] Akhand SS Liu Z Purdy SC Abdullah A Lin H Cresswell GM. Pharmacologic Inhibition of FGFR Modulates the Metastatic Immune Microenvironment and Promotes Response to Immune Checkpoint Blockade. Cancer Immunol Res (2020) 8:1542–53. doi: 10.1158/2326-6066.CIR-20-0235 PMC7710538 33093218

[242] Stacchiotti S Van Tine BA. Synovial Sarcoma: Current Concepts and Future Perspectives. J Clin Oncol (2018) 36:180–7. doi: 10.1200/JCO.2017.75.1941 29220290

[243] Kadoch C Crabtree GR. Reversible Disruption of mSWI/SNF (BAF) Complexes by the SS18-SSX Oncogenic Fusion in Synovial Sarcoma. Cell (2013) 153:71–85. doi: 10.1016/j.cell.2013.02.036 23540691 PMC3655887

[244] Jones KB Su L Jin H Lenz C Randall RL Underhill TM. SS18-SSX2 and the Mitochondrial Apoptosis Pathway in Mouse and Human Synovial Sarcomas. Oncogene (2013) 32:2365–71. doi: 10.1038/onc.2012.247 PMC3756901 22797074

[245] Saito T Nagai M Ladanyi M. SYT-SSX1 and SYT-SSX2 Interfere With Repression of E-Cadherin by Snail and Slug: A Potential Mechanism for Aberrant Mesenchymal to Epithelial Transition in Human Synovial Sarcoma. Cancer Res (2006) 66:6919–27. doi: 10.1158/0008-5472.CAN-05-3697 16849535

[246] Changchien Y-C Tátrai P Papp G Sápi J Fónyad L Szendrői M. Poorly Differentiated Synovial Sarcoma is Associated With High Expression of Enhancer of Zeste Homologue 2 (EZH2). J Transl Med (2012) 10:216. doi: 10.1186/1479-5876-10-216 23110793 PMC3494513

[247] Spurrell EL Fisher C Thomas JM Judson IR. Prognostic Factors in Advanced Synovial Sarcoma: An Analysis of 104 Patients Treated at the Royal Marsden Hospital. Ann Oncol (2005) 16:437–44. doi: 10.1093/annonc/mdi082 15653701

[248] Mir O Brodowicz T Italiano A Wallet J Blay J-Y Bertucci F. Safety and Efficacy of Regorafenib in Patients With Advanced Soft Tissue Sarcoma (REGOSARC): A Randomised, Double-Blind, Placebo-Controlled, Phase 2 Trial. Lancet Oncol (2016) 17:1732–42. doi: 10.1016/S1470-2045(16)30507-1 27751846

[249] Paoluzzi L Maki RG. Diagnosis, Prognosis, and Treatment of Alveolar Soft-Part Sarcoma: A Review. JAMA Oncol (2019) 5:254–60. doi: 10.1001/jamaoncol.2018.4490 30347044

[250] Flores RJ Harrison DJ Federman NC Furman WL Huh WW Broaddus EG. Alveolar Soft Part Sarcoma in Children and Young Adults: A Report of 69 Cases. Pediatr Blood Cancer (2018) 65:e26953. doi: 10.1002/pbc.26953 29350467

[251] Stockwin LH Vistica DT Kenney S Schrump DS Butcher DO Raffeld M. Gene Expression Profiling of Alveolar Soft-Part Sarcoma (ASPS). BMC Cancer (2009) 9:22. doi: 10.1186/1471-2407-9-22 19146682 PMC2635365

[252] Kummar S Allen D Monks A Polley EC Hose CD Ivy SP. Cediranib for Metastatic Alveolar Soft Part Sarcoma. J Clin Oncol (2013) 31:2296–302. doi: 10.1200/JCO.2012.47.4288 PMC3677840 23630200

[253] Stacchiotti S Mir O Le Cesne A Vincenzi B Fedenko A Maki RG. Activity of Pazopanib and Trabectedin in Advanced Alveolar Soft Part Sarcoma. Oncologist (2018) 23:62–70. doi: 10.1634/theoncologist.2017-0161 28754721 PMC5759809

[254] Reichardt P Lindner T Pink D Thuss-Patience PC Kretzschmar A Dörken B. Chemotherapy in Alveolar Soft Part Sarcomas. What do We Know? Eur J Cancer Oxf Engl 1990 (2003) 39:1511–6. doi: 10.1016/s0959-8049(03)00264-8 12855256

[255] Topalian SL Taube JM Pardoll DM. Neoadjuvant Checkpoint Blockade for Cancer Immunotherapy. Science (2020) 367. doi: 10.1126/science.aax0182 PMC7789854 32001626

